# Group contribution and atomic contribution models for the prediction of various physical properties of deep eutectic solvents

**DOI:** 10.1038/s41598-021-85824-z

**Published:** 2021-03-23

**Authors:** Reza Haghbakhsh, Sona Raeissi, Ana Rita C. Duarte

**Affiliations:** 1 LAQV, REQUIMTE, Departamento de Química da Faculdade de Ciências e Tecnologia, Universidade Nova de Lisboa, 2829-516 Caparica, Portugal; 2 School of Chemical and Petroleum Engineering, Shiraz University, Mollasadra Ave., 71348-51154 Shiraz, Iran

**Keywords:** Chemical engineering, Physical chemistry, Theoretical chemistry

## Abstract

The urgency of advancing green chemistry from labs and computers into the industries is well-known. The Deep Eutectic Solvents (DESs) are a promising category of novel green solvents which simultaneously have the best advantages of liquids and solids. Furthermore, they can be designed or engineered to have the characteristics desired for a given application. However, since they are rather new, there are no general models available to predict the properties of DESs without requiring other properties as input. This is particularly a setback when screening is required for feasibility studies, since a vast number of DESs are envisioned. For the first time, this study presents five group contribution (GC) and five atomic contribution (AC) models for densities, refractive indices, heat capacities, speeds of sound, and surface tensions of DESs. The models, developed using the most up-to-date databank of various types of DESs, simply decompose the molecular structure into a number of predefined groups or atoms. The resulting *AARD%* of densities, refractive indices, heat capacities, speeds of sound and surface tensions were, respectively, 1.44, 0.37, 3.26, 1.62, and 7.59% for the GC models, and 2.49, 1.03, 9.93, 4.52 and 7.80% for the AC models. Perhaps, even more importantly for designer solvents, is the predictive capability of the models, which was also shown to be highly reliable. Accordingly, very simple, yet highly accurate models are provided that are global for DESs and needless of any physical property information, making them useful predictive tools for a category of green solvents, which is only starting to show its potentials in green technology.

## Introduction

Technology has made intercontinental travel commonplace, giving humans a false sense of the planet; that it is all at their fingertips, that it is theirs to conquer and do as they please. However, the recent crisis of COVID-19, devastating populations and economies throughout all continents, was, and still is, an urgent wake-up call. The Earth is far more dominant than we have made ourselves believe, and it will continue to revive itself, with or without the human race. A recent note in *Nature* celebrates the 100th birthday of James Lovelock, the independent scientists who proposed the hypothesis known as “*Gaia*” (ancient Greek for *Earth*), which states that the planet is not a mere interstellar body inhabited by various life forms, but a vast self-regulating organism that regulates its destiny by corrective measures^1^. Along this path, the most intelligent form of life known so far, is perhaps more vulnerable to its own unthinking actions than previously understood. It is due time to take responsible actions regarding the health of the planet as a whole, and our own species as a part of it. This requires serious action from a multitude of approaches.

Green chemistry, which aims to reduce or eliminate the use or generation of hazardous material in the design of chemical products and processes, is one of the effective approaches that can be taken. With the workhorses of many of the industries being their solvents, research followed by implementation of green solvents into the industries can have profound impact. This can prevent the release of huge amounts of volatile organic compounds into the atmosphere.

The Deep Eutectic Solvents (DESs), introduced in 2003 by Abbott et al.^2^, are novel types of green solvents with a multitude of very unique properties. The majority of DESs simultaneously have the advantages of solids and liquids. The liquid phase is by far the preferred phase in the industries over gases that have issues of storage, leakage, and safety, and solids that have handling difficulties in continuous processes. However, when it comes to environmental concerns, solids are far more advantageous over liquids, as they pose either no threats of release into the atmosphere, or insignificant amounts even if they do. The highly desirable property of most DESs is that they are liquid, yet they release very little vapors to the atmosphere, making them ideal solvents in this respect. A second extremely exciting characteristic of a DES is that it is a designer solvent, i.e., it can be “engineered” to be what we require of it. In other words, a DES can be designed and tuned to have the specific characteristics that we need in a particular task. This is because a DES is the resulting mixture of two or more components, a Hydrogen Bond Acceptor (HBA), and a Hydrogen Bond Donor (HBD), which form a eutectic mixture. The appropriate choice of the HBA, HBD, and the corresponding ratios of these components among a vast list of choices allows for the desired tunability to the specific desired properties. A third great advantage of DESs is that they have the potential to be used in a multitude of applications. This is evidenced by the wide range of industrial prospects already suggested for DESs in the short time since they have been introduced to the scientific community, ranging from pharmaceuticals to the energy and environment sectors^3–13^. In fact, the number of research articles on DES, with due reason, has grown exponentially since their beginning. While 13 articles were indexed by Scopus in 2009, the number has grown by nearly 70-fold within one decade, with 2019 witnessing 886 scientific papers on DESs.

However, disregarding the type of envisioned application, one issue remains a common obstacle in nearly all of the endeavors: information on the physical properties of DESs. No process can be properly designed or optimized without accurate knowledge of the physical properties. However, alongside the vital necessity of physical property data for DESs, there are two major issues causing limitations. First is that DESs have been introduced only recently. Much of the properties have not yet been measured experimentally, and so, are unavailable when required. Second is the nature of DESs. In contrast to conventional solvents which are pure substances and rather limited in number, and so manageable, DESs are mixtures. They can be created from a multitude of HBA and HBD components, resulting in huge numbers of possible DESs. Experimental determination of the various properties of the immeasurable number of DESs is practically impossible. One possible solution to these issues is the modeling approach.

With the availability of predictive thermodynamic models, no time and no costs need to be endured to obtain the required physical properties. The importance of this issue is of an entirely different order for DESs. This is because DESs have the specific characteristic of being designer solvents, to be engineered among numerous choices. Therefore, feasibility studies can be a main aspect of well thought-through research on DES. Instead of a trial-and-error approach of random DESs, a proficient researcher would make informative choices. For this purpose, it is vital to have tools to allow the prediction of the properties required for feasibility studies, without actually stepping into the labs for making the DESs and measuring their physical properties. Such predictive tools can be indispensable for the success of various projects on DESs.

Despite this, up to now, there are no general thermodynamic models available which can directly estimate a desired physical property without requirement of other properties of the DES. One category of modeling studies in the literature involves equations of state^14–17^, which require knowledge of the critical properties and acentric factor. A number of correlations are also available, some being component specific only, so applicable to only the one specific DES that the constants of the model were optimized for^18,19^. Such correlations are not general, and so, not suitable for predictions or feasibility studies. Another category of correlations is also available which require the critical properties as their input^20–25^. Black-box computer models based on artificial intelligence have also been proposed in this regard. These are also correlative tools which are valid only for the investigated data bank^26–28^, and so, limited in their use.

One quite general and useful approach to physical property estimations is the group contribution (GC) technique. In the GC procedure, the structure of a compound is divided into a number of groups with predetermined weights. The summation of these weights is the only input parameter to the mathematical expression that estimates the desired property of the compound. GC models are reliable and very commonly-used by researchers to estimate various properties of compounds^29–31^. They also have been vastly used for the estimation of the critical properties of ionic liquids^32–34^. A very simplified form of the GC model is one where the constituent atoms are simply considered instead of functional groups of atoms. Such models are more specifically called atomic contribution (AC) models.

In this study, for the first time in literature, we follow the group contribution approach for the estimation of physical properties of DESs, to investigate whether this viewpoint will be applicable to DESs. Both the atomic contribution (AC) and the group contribution (GC) approaches are investigated. The physical properties of densities, refractive indices, heat capacities, speeds of sound and surface tensions are considered. The proposed models are developed on the most up-to-date databank of DESs^35–116^, encompassing the data available on DESs up to end of 2019.

## Results

Equations 1 to 30 in Table 1 present the developed GC and AC models. All of the investigated properties are functions of temperature, thus *T* introduces the system temperature to the equations, in kelvins. The superscripts of *G* and *A* denote the type of the model, being either GC or AC, respectively. *Mw* is the molecular weight of the DES in g/mol. $\Delta X_{1,i}$ and $\Delta X_{2,i}$ (where *X* = *ρ, n, Cp, u* or *σ*) are the contributions (weights) of each group/atom of type *i* for the GC and AC models. *k*_*i*_ and *l*_*j*_ indicate the number of occurrence of the functional group/atom of type *i* in the HBA and HBD molecules, respectively. *p* is the total number of HBA functional groups/atoms and *q* is the total number of HBD functional groups/atoms. *m*_*HBA*_ and *m*_*HBD*_ are the normalized number of moles of the HBA and HBD components making up the desired DES. For this purpose, the values of *m*_*HBA*_ and *m*_*HBD*_ are normalized based on the smallest value of *m*_*HBA*_ or *m*_*HBD*_ in any DES. For example, if for an arbitrary DES, the number of moles of HBA and HBD are 2 and 3, respectively, they should both be normalized by dividing each by 2 (which is the smaller number of moles), leading to the values of *m*_*HBA*_ = 1 and *m*_*HBD*_ = 1.5. These normalized values must be used in Eqs. 1 to 30. In this way, when using the proposed models, one of the HBA or HBD mole numbers will always be equal to one, while the other will be greater than one. The proposed models were developed based on data at atmospheric pressure, therefore, they are not recommended at higher or lower pressures.

**Table 1:** The list of proposed AC and GC models for density, refractive index, heat capacity, speed of sound and surface tension of DESs at atmospheric pressure.

Property	Model	
	GC model	
Density (g/cm^3^)	$\rho_{{_{1} }}^{G} = m_{HBA} \sum\limits_{i = 1}^{p} {k_{i} (\Delta \rho_{{_{1,i} }}^{G} )_{HBA} } + m_{HBD} \sum\limits_{i = 1}^{q} {l_{i} (\Delta \rho_{{_{1,i} }}^{G} )_{HBD} }$	(1)
	$$\rho_{2}^{G} = m_{HBA} \sum\limits_{i = 1}^{p} {k_{i} (\Delta \rho_{{_{2,i} }}^{G} )_{HBA} } + m_{HBD} \sum\limits_{i = 1}^{q} {l_{i} (\Delta \rho_{{_{2,i} }}^{G} )_{HBD} }$$	(2)
	$$\rho^{G} = \left( {\frac{{\rho_{1}^{G} }}{Mw}} \right)^{ - 0.2045} + \left( {\frac{{\rho_{2}^{G} }}{Mw}} \right)T^{ - 0.6785} + 0.2818$$	(3)
Refractive index	$n_{{_{1} }}^{G} = m_{HBA} \sum\limits_{i = 1}^{p} {k_{i} (\Delta n_{{_{1,i} }}^{G} )_{HBA} } + m_{HBD} \sum\limits_{i = 1}^{q} {l_{i} (\Delta n_{{_{1,i} }}^{G} )_{HBD} }$	(4)
	$$n_{2}^{G} = m_{HBA} \sum\limits_{i = 1}^{p} {k_{i} (\Delta n_{{_{2,i} }}^{G} )_{HBA} } + m_{HBD} \sum\limits_{i = 1}^{q} {l_{i} (\Delta n_{{_{2,i} }}^{G} )_{HBD} }$$	(5)
	$$n^{G} = \left( {n_{1}^{G} } \right)^{ - 0.3597} + \left( {n_{2}^{G} } \right)T^{ - 1.8254} + 1.3695$$	(6)
Heat capacity (J/mol K)	$Cp_{{_{1} }}^{G} = m_{HBA} \sum\limits_{i = 1}^{p} {k_{i} (\Delta Cp_{{_{1,i} }}^{G} )_{HBA} } + m_{HBD} \sum\limits_{i = 1}^{q} {l_{i} (\Delta Cp_{{_{1,i} }}^{G} )_{HBD} }$	(7)
	$$Cp_{2}^{G} = m_{HBA} \sum\limits_{i = 1}^{p} {k_{i} (\Delta Cp_{{_{2,i} }}^{G} )_{HBA} } + m_{HBD} \sum\limits_{i = 1}^{q} {l_{i} (\Delta Cp_{{_{2,i} }}^{G} )_{HBD} }$$	(8)
	$$Cp^{G} = \left( {Cp_{1}^{G} } \right)^{0.8653} + \left( {Cp_{2}^{G} } \right)T^{ - 0.4528} + 341.4081$$	(9)
Speed of sound (m/s)	$u_{{_{1} }}^{G} = m_{HBA} \sum\limits_{i = 1}^{p} {k_{i} (\Delta u_{{_{1,i} }}^{G} )_{HBA} } + m_{HBD} \sum\limits_{i = 1}^{q} {l_{i} (\Delta u_{{_{1,i} }}^{G} )_{HBD} }$	(10)
	$$u_{2}^{G} = m_{HBA} \sum\limits_{i = 1}^{p} {k_{i} (\Delta u_{{_{2,i} }}^{G} )_{HBA} } + m_{HBD} \sum\limits_{i = 1}^{q} {l_{i} (\Delta u_{{_{2,i} }}^{G} )_{HBD} }$$	(11)
	$$u^{G} = \left( {u_{1}^{G} } \right) - \left( {u_{2}^{G} } \right)T^{0.1851} + 1829.8799$$	(12)
Surface tension (mN/m)	$\sigma_{{_{1} }}^{G} = m_{HBA} \sum\limits_{i = 1}^{p} {k_{i} (\Delta \sigma_{{_{1,i} }}^{G} )_{HBA} } + m_{HBD} \sum\limits_{i = 1}^{q} {l_{i} (\Delta \sigma_{{_{1,i} }}^{G} )_{HBD} }$	(13)
	$$\sigma_{2}^{G} = m_{HBA} \sum\limits_{i = 1}^{p} {k_{i} (\Delta \sigma_{{_{2,i} }}^{G} )_{HBA} } + m_{HBD} \sum\limits_{i = 1}^{q} {l_{i} (\Delta \sigma_{{_{2,i} }}^{G} )_{HBD} }$$	(14)
	$$\sigma^{G} = \left( {\sigma_{1}^{G} } \right) - \left( {\sigma_{2}^{G} } \right)T^{0.0115} + 40.8235$$	(15)
**AC model**		
Density (g/cm^3^)	$\rho_{{_{1} }}^{A} = m_{HBA} \sum\limits_{i = 1}^{p} {k_{i} (\Delta \rho_{{_{1,i} }}^{A} )_{HBA} } + m_{HBD} \sum\limits_{i = 1}^{q} {l_{i} (\Delta \rho_{{_{1,i} }}^{A} )_{HBD} }$	(16)
	$$\rho_{2}^{A} = m_{HBA} \sum\limits_{i = 1}^{p} {k_{i} (\Delta \rho_{{_{2,i} }}^{A} )_{HBA} } + m_{HBD} \sum\limits_{i = 1}^{q} {l_{i} (\Delta \rho_{{_{2,i} }}^{A} )_{HBD} }$$	(17)
	$$\rho^{A} = \left( {\frac{{\rho_{1}^{A} }}{Mw}} \right)^{ - 0.4093} + \left( {\frac{{\rho_{2}^{A} }}{Mw}} \right)T^{ - 0.7434} + 0.5139$$	(18)
Refractive index	$n_{{_{1} }}^{A} = m_{HBA} \sum\limits_{i = 1}^{p} {k_{i} (\Delta n_{{_{1,i} }}^{A} )_{HBA} } + m_{HBD} \sum\limits_{i = 1}^{q} {l_{i} (\Delta n_{{_{1,i} }}^{A} )_{HBD} }$	(19)
	$$n_{2}^{A} = m_{HBA} \sum\limits_{i = 1}^{p} {k_{i} (\Delta n_{{_{2,i} }}^{A} )_{HBA} } + m_{HBD} \sum\limits_{i = 1}^{q} {l_{i} (\Delta n_{{_{2,i} }}^{A} )_{HBD} }$$	(20)
	$$n^{A} = \left( {n_{1}^{A} Mw} \right)^{ - 0.2975} + \left( {n_{2}^{A} Mw} \right)T^{ - 2.9213} + 1.4335$$	(21)
Heat capacity (J/mol K)	$Cp_{{_{1} }}^{A} = m_{HBA} \sum\limits_{i = 1}^{p} {k_{i} (\Delta Cp_{{_{1,i} }}^{A} )_{HBA} } + m_{HBD} \sum\limits_{i = 1}^{q} {l_{i} (\Delta Cp_{{_{1,i} }}^{A} )_{HBD} }$	(22)
	$$Cp_{2}^{A} = m_{HBA} \sum\limits_{i = 1}^{p} {k_{i} (\Delta Cp_{{_{2,i} }}^{A} )_{HBA} } + m_{HBD} \sum\limits_{i = 1}^{q} {l_{i} (\Delta Cp_{{_{2,i} }}^{A} )_{HBD} }$$	(23)
	$$Cp^{A} = \left( {Cp_{1}^{A} } \right)^{0.5592} + \left( {Cp_{2}^{A} } \right)T^{0.7325} + 31.7092$$	(24)
Speed of sound (m/s)	$u_{{_{1} }}^{A} = m_{HBA} \sum\limits_{i = 1}^{p} {k_{i} (\Delta u_{{_{1,i} }}^{A} )_{HBA} } + m_{HBD} \sum\limits_{i = 1}^{q} {l_{i} (\Delta u_{{_{1,i} }}^{A} )_{HBD} }$	(25)
	$$u_{2}^{A} = m_{HBA} \sum\limits_{i = 1}^{p} {k_{i} (\Delta u_{{_{2,i} }}^{A} )_{HBA} } + m_{HBD} \sum\limits_{i = 1}^{q} {l_{i} (\Delta u_{{_{2,i} }}^{A} )_{HBD} }$$	(26)
	$$u^{A} = u_{1}^{A} - u_{2}^{A} T^{0.0258} + 1607.4690$$	(27)
Surface tension (mN/m)	$\sigma_{{_{1} }}^{A} = m_{HBA} \sum\limits_{i = 1}^{p} {k_{i} (\Delta \sigma_{{_{1,i} }}^{A} )_{HBA} } + m_{HBD} \sum\limits_{i = 1}^{q} {l_{i} (\Delta \sigma_{{_{1,i} }}^{A} )_{HBD} }$	(28)
	$$\sigma_{2}^{A} = m_{HBA} \sum\limits_{i = 1}^{p} {k_{i} (\Delta \sigma_{{_{2,i} }}^{A} )_{HBA} } + m_{HBD} \sum\limits_{i = 1}^{q} {l_{i} (\Delta \sigma_{{_{2,i} }}^{A} )_{HBD} }$$	(29)
	$$\sigma^{A} = \sigma_{1}^{A} - \sigma_{2}^{A} T^{0.0099} + 40.4052$$	(30)

For each of the investigated physical properties, Table 2 presents the contributions (weights) of the functional groups for the GC models, while Table 3 lists the corresponding values for the atoms in the AC models.

**Table 2:** The list of functional groups and their contributions for the GC models for density, refractive index, heat capacity, speed of sound and surface tension.

Group	Density		Refractive index		Heat capacity		Speed of sound		Surface tension	
	$\Delta \rho_{1}^{G}$	$\Delta \rho_{2}^{G}$	$\Delta n_{1}^{G}$	$\Delta n_{2}^{G}$	$\Delta Cp_{1}^{G}$	$\Delta Cp_{2}^{G}$	$\Delta u_{1}^{G}$	$\Delta u_{2}^{G}$	$\Delta \sigma_{1}^{G}$	$\Delta \sigma_{2}^{G}$
**Without Ring**										
$- CH_{3}$	61.91	1.53	160.09	5.70	85.44	− 445.78	381.49	164.90	19.99	8.31
$- CH_{2} -$	41.87	8.27	19.02	5.80	1.13	159.25	36.98	16.26	16.77	17.40
$> CH -$	− 7.03	− 29.59	− 40.22	107.99	139.93	− 22.20	174.57	47.75	17.73	17.65
$> C <$	− 156.89	53.40	− 138.77	310.05	434.91	39.84	41.85	− 146.00	12.04	22.76
$> C =$	− 194.64	313.70	− 225.17	328.86	–	–	40.33	6.31	22.57	11.56
$> C = O$	23.62	147.10	1168.38	620.49	–	–	–	–	11.93	25.86
$= CH_{2}$	− 67.50	− 41.90	25.75	91.21	–	–	–	–	25.50	23.71
$= CH -$	6.35	− 92.34	− 7.63	121.13	–	–	–	–	8.45	10.48
$O - C = O$	− 12.70	71.88	564.20	− 76.33	− 123.27	34.46	56.86	− 63.70	–	–
$- COOH$	− 21.79	73.88	28.67	− 46.23	38.00	− 529.75	196.99	61.88	11.82	8.62
$OH$	− 30.59	11.13	26.23	− 7.34	12.47	− 527.29	337.31	113.45	22.00	18.90
$- O -$	− 54.78	4.11	− 14.76	− 1.76	60.87	− 273.17	− 52.21	− 21.97	9.74	5.80
$\left[ { > N < } \right]^{ + }$	16.78	85.54	− 210.44	166.33	− 63.80	− 105.70	301.46	90.53	8.34	20.74
$> N - /\left[ { > NH - } \right]^{ + }$	− 19.15	− 53.08	− 15.08	44.23	− 79.15	− 10.19	–	–	19.97	15.89
$> NH/\left[ { > NH_{2} } \right]^{ + }$	145.55	22.74	− 218.94	128.78	–	–	–	–	10.93	9.47
$- NH_{2} /\left[ { - NH_{3} } \right]^{ + }$	− 8.21	32.36	101.73	124.03	− 1.06	− 217.59	190.29	36.42	17.42	14.97
$= NH$	− 91.68	148.78	− 172.92	169.16	–	–	–	–	14.14	19.14
$F$	− 5.99	23.58	− 119.61	208.64	− 4.23	− 146.36	52.48	46.58	18.15	10.91
$Cl$	4.39	17.68	68.23	− 16.50	− 92.23	− 88.93	254.11	− 12.96	11.69	19.83
$Br$	24.90	176.21	15.77	260.47	21.24	58.15	–	–	13.61	23.94
$P$	− 45.29	289.98	− 37.67	− 873.14	26.76	46.60	–	–	19.49	19.10
$I$	–	–	7.12	5.30	–	–	–	–	–	–
$- S -$	–	–	− 119.56	1.81	–	–	–	–	10.15	15.56
$O = S = O$	− 62.28	18.30	− 127.74	− 587.05	–	–	85.30	− 254.08	–	–
**Saturated Ring**										
$= CH -$	22.10	12.76	− 1.55	97.75	18.55	− 58.81	129.80	112.73	22.18	17.46
$> C =$	− 19.37	18.87	− 55.27	116.96	− 0.52	− 62.85	19.82	− 294.30	5.67	18.46
$= N -$	69.14	53.63	–	–	–	–	–	–	–	–
$\left[ { > N = } \right]^{ + }$	–	–	–	–	–	–	64.83	− 72.41	–	–
$> N - /\left[ { > NH - } \right]^{ + }$	104.13	235.50	–	–	–	–	46.44	− 75.25	–	–
$OH$	0.81	17.09	86.19	78.79	− 12.46	− 314.64	135.18	15.47	7.56	17.90
$Cl$	− 20.59	50.74	− 9.90	− 67.41	–	–	–	–	–	–
$- O -$	− 10.38	13.66	–	–	–	–	–	–	–	–
$O = S = O$	–	–	− 29.04	84.06	–	–	–	–	–	–
**Unsaturated Ring**										
$- CH_{2} -$	36.89	2.31	52.44	23.81	–	–	155.46	100.18	–	–
$> CH -$	47.49	187.89	338.04	− 185.99	25.90	− 95.14	142.09	− 20.32	16.20	17.72
$> C <$	114.87	544.91	− 191.20	803.92	117.51	25.39	− 104.84	14.41	15.57	19.85
$> C = O$	–	–	–	–	–	–	53.65	− 228.27	–	–
$- O -$	64.27	236.80	− 141.08	736.54	53.69	8.77	− 16.50	0.53	21.17	7.27
$> NH/\left[ { > NH_{2} } \right]^{ + }$	42.77	90.43	− 54.53	644.84	–	–	7.11	− 36.01	–	–
$OH$	51.89	186.16	− 77.33	453.43	103.46	− 74.96	29.90	60.11	11.27	6.89

**Table 3:** The list of functional groups and their contributions for the AC models for density, refractive index, heat capacity, speed of sound and surface tension.

Atom	Density		Refractive index		Heat capacity		Speed of sound		Surface tension	
	$\Delta \rho_{1}^{A}$	$\Delta \rho_{2}^{A}$	$\Delta n_{1}^{A}$	$\Delta n_{2}^{A}$	$\Delta Cp_{1}^{A}$	$\Delta Cp_{2}^{A}$	$\Delta u_{1}^{A}$	$\Delta u_{2}^{A}$	$\Delta \sigma_{1}^{A}$	$\Delta \sigma_{2}^{A}$
$H$	11.10	− 23.76	27.59	− 79.40	0.1043	0.0139	74.50	68.77	2.29	2.74
$C$	11.59	35.41	− 35.99	171.55	0.3838	0.0205	732.22	633.37	21.39	20.02
$N$	202.25	386.06	− 42.29	362.85	0.3431	0.0002	3445.83	2924.48	19.34	16.94
$O$	− 21.11	69.14	9.63	54.84	3.1802	0.1541	1858.40	1589.13	5.19	3.32
$S$	30.10	− 2.34	106.15	466.54	–	–	–	–	6.27	1.40
$P$	− 167.87	574.46	–	–	551.0351	0.0050	–	–	11.95	12.18
$F$	–	–	–	–	–	–	–	–	13.88	15.67
$Cl$	− 3.12	212.74	− 4.52	− 105.19	482.0574	0.8520	416.51	155.70	18.22	1.32
$Br$	63.22	123.77	− 92.16	− 80.13	548.3321	0.0050	–	–	19.36	1.46
$I$	–	–	− 103.53	− 18.40	–	–	–	–	–	–

The details of calculations by the proposed GC and AC models are provided in Appendix A in the Supporting Information, where the density, refractive index, heat capacity, speed of sound, and surface tension of two exemplary DES are calculated in a step-by-step procedure.

The deviation of each point is calculated according to Eq. (31) for the various properties,

31 $$ D = X_{{^{i} }}^{Model} - X_{i}^{Exp} $$

**Figure 1:**
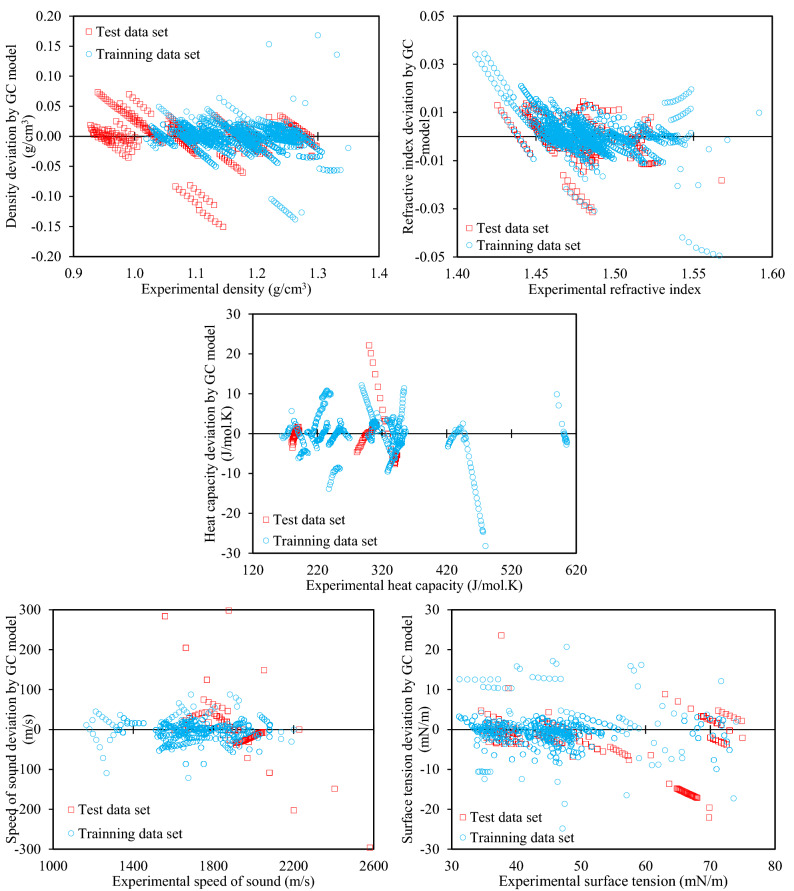
Deviations of the various physical properties from the experimental values for the training and test data sets by the proposed GC models.

**Figure 2:**
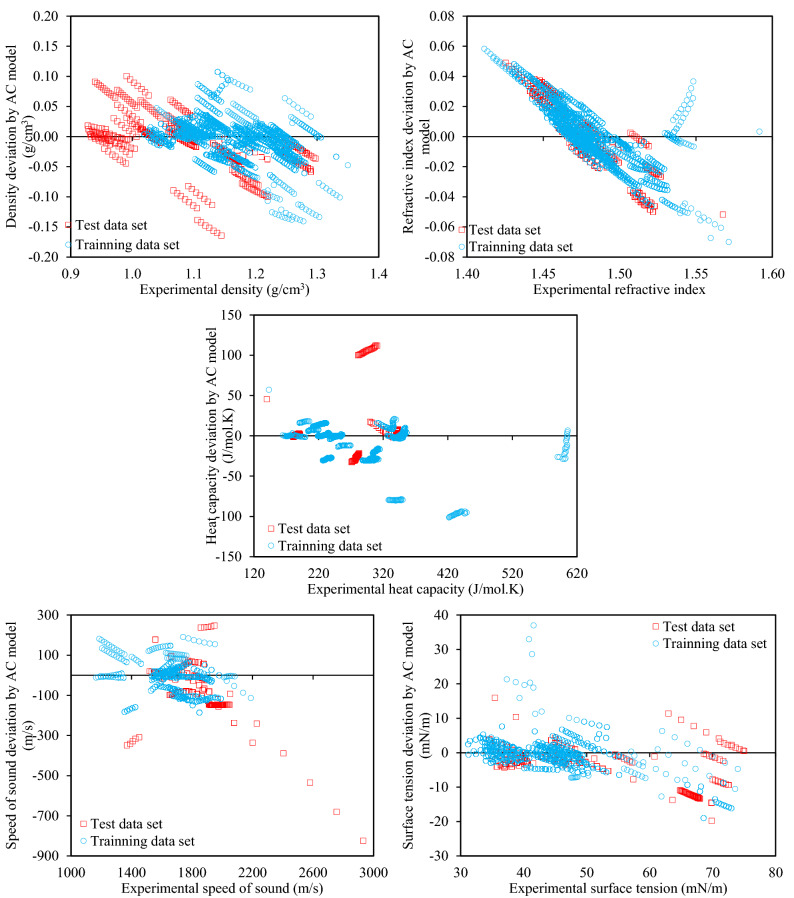
Deviations of the various physical properties from the experimental values for the training and test data sets by the proposed AC models.

The deviations are shown separately for the training and test datasets in Fig. 1 for the GC model, and Fig. 2 for the AC model. From these figures it is interpreted that for all of the properties, there are no significant differences between the training and test datasets in terms of deviations, as both sets cover similar ranges. This is quite promising for the accuracy of predictions, and holds for both the GC models (Fig. 1) and the AC models (Fig. 2).

Following the above validation of the test dataset using Figs. 1 and 2, all of the following discussions are for the entire databank, as we saw no necessity to separate the correlative and predictive datasets which show rather similar performances in accuracies.

The accuracies of the models are further investigated using the statistical parameters of absolute average relative deviation percent (*AARD%*), absolute relative deviation percent (*ARD%*), relative deviation percent (*RD%*), absolute average deviation (*AAD*), and standard deviation (*S*), as defined by Eqs. (32) – (36), respectively:

32 $$ AARD\% = \frac{100}{N}\sum\limits_{i = 1}^{N} {\left| {\frac{{X_{i}^{Model} - X_{i}^{Exp} }}{{X_{i}^{Exp} }}} \right|} $$

33 $$ ARD\% = 100\left| {\frac{{X_{i}^{Model} - X_{i}^{Exp} }}{{X_{i}^{Exp} }}} \right| $$

34 $$ RD\% = 100\left( {\frac{{X_{i}^{Model} - X_{i}^{Exp} }}{{X_{i}^{Exp} }}} \right) $$

35 $$ AAD = \frac{1}{N}\sum\limits_{i = 1}^{N} {\left| {X_{{^{i} }}^{Model} - X_{i}^{Exp} } \right|} $$

36 $$ S = \sqrt {\frac{{\sum\limits_{i = 1}^{N} {\left( {X_{{^{i} }}^{Model} - X_{i}^{Exp} } \right)}^{2} }}{N}} $$

In these equations, *N* is the number of investigated data points, $X_{{^{i} }}^{Model}$ is the calculated value of the property *X* by the model, and $X_{i}^{Exp}$ is the corresponding experimental value of the property *X*, where *X* can be *ρ, n, C*_*p*_*, u,* or *σ*.

The values of these statistical parameters for the entire dataset are presented in Table 4 for both the GC and AC models. The small deviations with respect to the experimental values indicate the accuracies of both models.

**Table 4:** The comparison for the calculated statistical parameters of GC, AC and literature models for density, refractive index, heat capacity, speed of sound and surface tension.

Property	Model	Number of investigated data	*AARD%*	*Maximum ARD%*	*AAD*	*S*
Density	AC	1239	2.49	42.79	0.03 g/cm^3^	0.05 g/cm^3^
	GC	1239	1.44	19.29	0.02 g/cm^3^	0.03 g/cm^3^
	Haghbakhsh et al.^25^	1239	3.12	18.00	0.03 g/cm^3^	0.05 g/cm^3^
	Rackett^120^	1239	17.01	109.32	0.22 g/cm^3^	0.30 g/cm^3^
	Spencer and Danner^121^	1239	27.72	281.68	0.32 g/cm^3^	0.46 g/cm^3^
	Mjalli et al.^18^	1239	27.37	279.43	0.31 g/cm^3^	0.45 g/cm^3^
Refractive index	AC	1117	1.03	4.46	0.01	0.02
	GC	1117	0.37	3.61	0.01	0.01
	Taherzadeh et al.^20^	1117	1.05	4.86	0.02	0.02
	Riazi and Daubert^122^	1117	11.09	47.32	0.13	0.21
	Riazi and Daubert^123^	1117	10.53	45.67	0.12	0.20
	Riazi and Al-Sahhaf^124^	1117	1.81	6.32	0.04	0.04
	Lorentz–Lorenz^125^	1117	1.98	7.01	0.05	0.05
Heat capacity	AC	461	9.93	47.48	34.42 J/mol K	58.63 J/mol K
	GC	461	3.26	47.60	8.87 J/mol K	21.31 J/mol K
	Taherzadeh et al.^23^	461	4.89	45.87	13.03 J/mol K	18.93 J/mol K
	Ahmadi et al.^126^	461	16.66	38.06	105.71 J/mol K	135.10 J/mol K
	Huang et al.^127^	461	55.24	74.12	170.11 J/mol K	188.07 J/mol K
	Ge et al.^128^	461	19.00	122.32	111.80 J/mol K	139.47 J/mol K
	Oster et al.^129^	461	16.51	116.59	106.00 J/mol K	131.74 J/mol K
Speed of sound	AC	398	4.52	78.50	117.19 m/s	144.99 m/s
	GC	398	1.62	20.05	28.64 m/s	59.37 m/s
	Peyrovedin et al.^24^	398	5.59	34.18	99.55 m/s	144.82 m/s
	Haghbakhsh et al.^130^	398	10.17	75.39	208.43 m/s	340.46 m/s
	Hekayati and Esmaeilzadeh^131^	398	9.19	36.43	175.98 m/s	224.38 m/s
	Gardas and Coutinho^132^	398	9.27	31.98	183.66 m/s	238.12 m/s
	Singh and Singh^133^	398	40.04	96.91	708.20 m/s	863.87 m/s
Surface tension	AC	538	7.80	88.97	3.84 mN/m	6.29 mN/m
	GC	538	7.59	62.35	3.75 mN/m	6.07 mN/m
	Haghbakhsh et al.^22^	538	8.61	59.67	4.51 mN/m	6.95 mN/m
	Escobedo and Mansoori^134^	538	79.92	90.37	38.17 mN/m	39.71 mN/m
	Curl and Pitzer^135^	538	16.51	162.40	9.52 mN/m	12.80 mN/m
	Gharagheizi et al.^136^	538	22.45	57.17	11.50 mN/m	14.16 mN/m

According to the results, the GC models show smaller error values for almost all of the statistical parameters in comparison to the AC model. The greatest differences in accuracies between the two models are observed for refractive index, heat capacity, and speed of sound, which have GC *AARD%* values that are nearly one-third of the corresponding AC models. For density, GC still show less errors than AC, while for surface tension, both models have nearly the same errors, being only marginally lower for GC. The better performances of GC models are not surprising. Functional groups are the units of calculations in the GC models, while the AC models break the units down to individual atoms. Functional groups can be strong indicators of the nature, and hence properties of compounds. While a GC functional group can distinguish between, for example, acids and alcohols, in the AC models a hydrogen atom behaves the same whether in a hydrocarbon, an acid, or an alcohol. Chemistry, of course, has taught us the significant differences in the behavior of the H atom within CH_3_ and OH. This highlights the main preference of the GC models over the AC models. However, the AC models of this study, although less accurate, are still acceptable in their errors and can be used not only for estimations, but also predictions. The AC models have the main advantage of simplicity. Decomposition of a compound into its atoms is, in fact, so simple that it allows an atomic model to be very easily incorporated into computer codes and software. This is not as easily done for the GC models. Furthermore, the decomposition into atoms always gives a unique result for a specific structure, while the decomposition into groups can sometimes be up to different interpretations, leading to different results. Because both models have acceptable results and errors, this double-model study allows a freedom of choice by the users depending on their aims, circumstances, and desired accuracy.

For a more detailed investigation, the values of *AARD%* and *maximum ARD%* are also presented individually for each specific DES in Tables S1–S5 for both the GC and AC models.

Furthermore, to check the distribution of errors over the entire range, the number of data points corresponding to their *ARD%* values were categorized into four *ARD%* ranges and reported in Table 5. According to the previously deduced results of Table 4 that the GC models have the lower errors, it is expected that the GC models will have a greater number of data in the lower *ARD%* ranges with respect to the AC models. This is validated by Table 5. For all five physical properties the GC models have the greatest number of data points within the smallest *ARD%* category. In the AC models, however, the data are more evenly distributed throughout the various error categories, although still having the greatest number of data within the least erroneous category. This holds for all of the properties.

**Table 5:** The distributions of the calculated values of *ARD%* of the GC and AC models for density, refractive index, heat capacity, speed of sound and surface tension.

Property	*ARD%* range	AC	GC
Density	< 2%	719	993
	2–4%	292	160
	4–6%	93	40
	> 6%	135	46
	Total	1239	1239
Refractive index	< 1%	653	1043
	1–2%	295	57
	2–3%	121	12
	> 3%	48	5
	Total	1117	1117
Heat capacity	< 2%	168	323
	2–5%	57	102
	5–10%	95	11
	> 10%	141	25
	Total	461	461
Speed of sound	< 2%	162	307
	2–5%	77	71
	5–10%	122	12
	> 10%	37	8
	Total	398	398
Surface tension	< 5%	263	322
	5–10%	139	108
	10–15%	71	35
	> 15%	65	73
	Total	538	538

**Figure 3:**
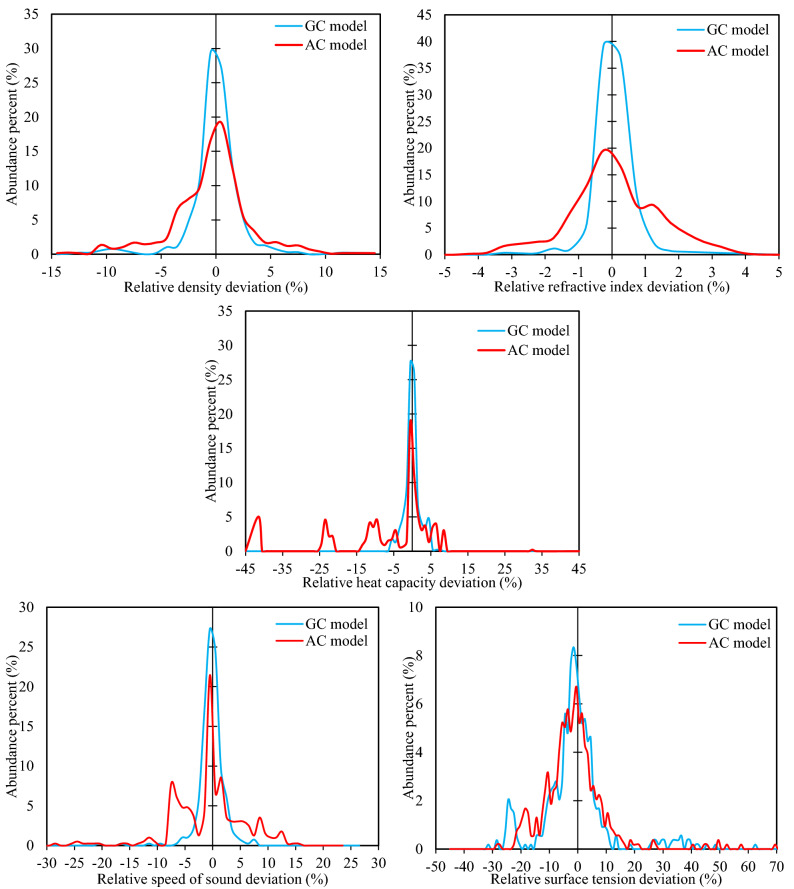
The comparisons for calculated relative deviation percentages of investigated physical properties for proposed GC and AC models for the entire dataset.

The distribution of the data into different ranges according to their *RD%* is shown graphically in Fig. 3. By differentiating between positive and negative relative deviations, this figure can indicate any possible bias regarding overestimations or underestimations, which could not be distinguished using the *ARD%* distribution comparison of Table 5. According to Fig. 3, both models show rather normal behavior in *RD%,* with no bias in their estimations, as the bell-shaped curves are more or less symmetric around the point zero. This holds for all of the different properties. Furthermore, the rather tall and slim shapes of the *RD*% domes are evidences of the high accuracy of the property models for the majority of the data, as contrasted to the more flattened-out shapes, which would have resulted if the accuracies were not high for a larger number of data. It is further observed in Fig. 3 that the peaks of the GC models are situated higher than the corresponding AC peaks, indicating the more reliable results of the GC model for a greater number of DESs.

Table 6 presents the results of the two models based on the molecular weights of the DESs, categorized into four groups (molecular weight ranges: < 100, 100–150, 150–200, and > 200). While some group contribution methods of literature show systematic changes of errors with increasing molecular weights, this is not the case with the GC model of this study for any of the properties. In the case of the AC model, however, greater errors are observed for the larger molecular weights for the properties of heat capacity and speed of sound.

**Table 6:** Comparison of errors (*AARD%*) according to the molecular weights of the DESs for the GC and AC models.

Molecular weight range of DESs	AARD%		Number of data points
	GC	AC	
**Density**			
80–100	1.62	1.91	202
100–150	1.36	2.50	643
150–200	1.55	2.99	300
200–267	1.28	2.09	94
**Refractive index**			
69–100	0.49	1.09	167
100–150	0.31	0.91	522
150–200	0.37	1.54	271
200–267	0.47	0.46	157
**Heat capacity**			
75–100	2.95	3.70	70
100–150	4.80	8.56	234
150–200	0.78	12.63	92
200–235	1.58	17.69	65
**Speed of sound**			
87–100	1.63	2.73	82
100–150	1.40	4.41	210
150–191	2.04	6.01	106
**Surface tension**			
69–100	4.69	5.80	78
100–150	9.52	9.19	208
150–200	4.97	9.37	134
200–267	8.86	3.81	118

The performances of the models are further investigated according to the nature of the HBA and HBD constituents and the comparisons are presented in Tables S6 and S7 of the Supporting Information.

All of the models of this study are also compared to the available literature models on DESs for each property (Table 4). It should be noted that component-specific literature models were not considered in this comparison, i.e., correlations developed for only a specific DES with equation constants that are valid for that one particular DES only. However, for a more comprehensive investigation, since models specific to DESs are very limited, we have also considered correlations for a close family of solvents, i.e., the ionic liquids. Additionally, physical property models for organic compounds have also been considered for a broader comparison. Table 4 presents these results for each of the investigated physical properties.

Regarding density, the only available generalized DES model of literature is the correlation of Haghbakhsh et al.^25^. The GC and AC models of this work show lower *AARD*% values, and almost similar values of *AAD* and *S* as the correlation of Haghbakhsh et al.^25^. However, a further issue of importance, in addition to accuracy, is the wide applicability and simplicity of a model. The correlation of Haghbakhsh et al.^25^ has the following functionality.

37 $$ \rho = - 1.13 \times 10^{ - 6} T_{c}^{2} + 2.566 \times 10^{ - 3} T_{c} + 0.2376\omega^{0.2211} - 4.67 \times 10^{ - 4} V_{c} - 4.64 \times 10^{ - 4} T $$

This temperature-dependent function requires the critical temperature (*T*_*c*_), critical volume (*V*_*c*_*)*, and acentric factor (*ω*) of the DES. These properties, when not available, can be calculated by the modified Lydersen-Joback–Reid group contribution model for each of the HBA and HBD components^117,118^, followed by the use of an appropriate mixing rule, such as the Lee-Kesler mixing rules^119^ to calculate the desired property for the DES. In this manner, the calculations of the input parameters, alone, require nine different calculations, six of which are themselves group contribution in nature. The calculations required by the model of this study are far less cumbersome. In addition to the models of Haghbakhsh et al.^25^ and Mjalli et al.^18^ specific to DESs, the general density correlations of Rackett^120^, Spencer and Danner^121^ were compared to the proposed GC/AC models of this study. In general, the present GC and AC models are both superior not only to the Rackett^120^, Spencer and Danner^121^ models, which are general, but even the correlations developed specifically for DESs.

In the literature, there is only one generalized model available for the refractive indices of DESs, as given by Taherzadeh et al.^20^

38 $$ n_{D} = 5.17 \times 10^{ - 2} \omega^{3} - 11.625\frac{{\omega^{2} }}{Mw} + 2.27 \times 10^{ - 3} P_{c} + 1.3668 + \frac{25.89\omega }{T} $$

The results, compared in Table 4, indicate that the GC approach outperforms the other two. The AC model shows slightly better results than those of Taherzadeh et al.^20^ Since the literature model requires knowledge of the critical pressure and acentric factor, which are themselves calculated by a combination of other group contribution models and mixing rules^117–119^, the two models of this work are, not only higher in accuracy, but also easier in calculations. Furthermore, results of two models by Riazi and Daubert^122,123^ as well as the models of Riazi and Al-Sahhaf^124^ and Lorentz–Lorenz^125^, all developed generally for organic compounds, are compared in Table 4. The results indicate that the Riazi and Al-Sahhaf^124^ and Lorentz–Lorenz^125^ models are promising models for DESs, however, both of the proposed GC/AC models still outperform the former.

Apart from the model proposed in this study, there is one further generalized model available in the literature for calculating the heat capacities of DESs, as proposed by Taherzadeh et al.^23^,

39 $$ C_{p} = 3.8 \times 10^{ - 4} \frac{{Mw^{3} }}{{P_{c}^{6} }} + 6.3 \times 10^{ - 5} Mw^{2\omega } - \frac{24577.4}{{Mw}} - 94.9 + 132.27T^{{{\raise0.7ex\hbox{$1$} \!\mathord{\left/ {\vphantom {1 4}}\right.\kern-\nulldelimiterspace} \!\lower0.7ex\hbox{$4$}}}} - 2.911V_{c} + 2514.2 $$

Table 4 compares the results, indicating the GC model to have superior accuracy than either the AC model or the model of Taherzadeh et al.^23^ This is the case for all of the statistical parameters investigated. The model of Taherzadeh et al.^23^ shows better results than the AC model, at the cost of more cumbersome calculations. The AC model is easier to use than both the models of Taherzadeh et al. and GC. In addition to the model of Taherzadeh et al.^23^, the literature models for the next closest families of substances were considered in the comparisons. These include the heat capacity correlations of Ahmadi et al.^126^, Huang et al.^127^, Ge et al.^128^ and Oster et al.^129^ which were developed for ionic liquids (Table 4), indicating that none of the heat capacity models proposed for ionic liquids are suitable.

For comparison with DES literature models on the speed of sound, only one general correlation was available, namely the approach of Peyrovedin et al.^24^,

40 $$ u = \omega \left[ {7.378Mw - 2.012T} \right] - 2.911V_{c} + 2514.2 $$

According to the results given in Table 4, the GC model shows higher accuracies with respect to all of the statistical parameters investigated. Following the GC, the AC model shows the better *AARD%* value with respect to the DES model of Peyrovedin et al^24^. The GC/AC models also show better results with respect to the ionic liquid-specific models of Haghbakhsh et al.^130^, Hekayati and Esmaeilzadeh^131^, Gardas and Coutinho^132^ and Singh and Singh^133^.

The literature correlation of Haghbakhsh et al.^22^, specifically developed for the surface tension of DESs, has the following functionality.

41 $$ \sigma = \begin{array}{*{20}c} {393.4} \\ \end{array} Ln(\rho )\begin{array}{*{20}c} { - 5.3 \times 10^{ - 5} } \\ \end{array} \omega^{{P_{c} }} \begin{array}{*{20}c} { - 3.72 \times 10^{ - 2} } \\ \end{array} T_{c} Ln\left( {\rho^{2} \left[ {V_{c} - \frac{{\begin{array}{*{20}c} {50.3} \\ \end{array} }}{{\omega^{2} }}} \right]} \right) + \frac{{\begin{array}{*{20}c} {1.132} \\ \end{array} Mw\sqrt T }}{{P_{c} Ln\left( {\frac{{V_{c} \rho }}{{\sqrt {T_{c} } }}} \right)}} + \begin{array}{*{20}c} {108.9} \\ \end{array} $$

Table 4 shows that the GC model has the smallest statistical errors in all aspects, and so it is the most reliable of the three. Following the GC, the AC model is more accurate than the model of Haghbakhsh et al.^22^ The AC model is the simplest of the three models, and the model of Haghbakhsh et al.^22^ requires the greatest amount of calculations since the values of critical temperature, critical pressure, critical volume and acentric factor, when not available, need to be calculated by other group contribution methods^117,118^ and mixing rules^119^, in addition to the calculation of density by the DES model^25^. Also, both the GC/AC models show better results with respect to the organic compound models of Escobedo and Mansoori^134^, Curl and Pitzer^135^ and Gharagheizi et al.^136^, which is of course expected as these are more generalized models.

## Discussion

Up to date, there are no direct group contribution models available in the literature to estimate a variety of physical properties of DESs of various types and natures in order to fill this vital gap, we decided to propose two models, a group contribution model and an atomic contribution model for the estimation of some of the most important physical properties of DESs. In order to cover the properties of density, refractive index, heat capacity, speed of sound and surface tension. The methods presented are general and applicable to a great range of DESs. This is not only because a large number of the groups or atoms of DESs are covered, but also because the databank used to develop the models is the most recent and complete set of data to date. Furthermore, because the group contribution models consider the effects of different functional groups, they are also predictive models, possessing the physical backgrounds of group contribution models. Therefore, with the current exponential growth of academic and industrial interest in DESs, the models provided in this study can be of significant value for the estimation of physical properties which are often necessary in the progress of the field of DESs.

With both the group and atomic contribution models, our goal was simplicity of the groups for ease of use. For this reason, the number of groups of the model is rather small compared to typical group contribution models, and the groups, themselves, are quite simple. Because of this, we expect that users will not be confronted with the ambiguities and doubts, and even multiple structural decomposition possibilities that often occur when using literature GC methods.

In order to develop the models, the most complete experimental data bank up to date was gathered from literature. This includes 1239, 117, 461, 398 and 538 data points from 149, 142, 24, 37 and 98 DESs, for density, refractive index, heat capacity, speed of sound and surface tension, respectively. Each databank was divided randomly into the two groups of training (70–80%) and testing (30–20%) data sets.

An extensive and comprehensive statistical investigation of errors was carried out on the developed GC and AC models. The results were shown to be quite accurate for all of the properties, with the GC model being superior to the AC model regarding errors. In brief, the calculated values of *AARD%* for the proposed GC models were 1.44, 0.37, 3.26, 1.62 and 7.59% for density, refractive index, heat capacity, speed of sound and surface tension, respectively. The corresponding values for the AC models were 2.49, 1.03, 9.93, 4.52 and 7.80%. Such results are not surprising because the GC models break the molecular structure into groups, whereas the AC models divide them simply into atoms. Therefore, if the chemical formula of two or more different components are the same (for example glucose, fructose and mannose as the HBD), the AC models cannot differentiate among them, while the GC models can. The AC models are also unable to distinguish among isomers. By proposing both AC and GC models in this study, we have provided the freedom of choice between greater simplicity or higher accuracy, depending on the aims and needs and limitations of the users. The choice can therefore be different in different cases.

For all of the physical properties covered in this work, the proposed GC models showed greater accuracy than the available literature correlations. However, the proposed AC models, while being more reliable than the literature correlations for density, refractive index, and surface tension, had less accuracy in the cases of heat capacity and speed of sound.

To summarize the pros and cons of the models proposed here in comparison to those available in literature for the estimation of DES physical properties, we point to the following. With respect to the literature correlations for DESs, they are either component-specific models, or else they have been developed for very limited numbers of DESs, and so are not widely-applicable to all types of DES families. For each property of density, refractive index, heat capacity, speed of sound and surface tension, there is only on global DES model available so far in open literature, each of which has been compared here in detail by providing numeric results of their errors. These generalized literature correlations for DESs are worthy in their own right, however the models presented here can be considered preferable due to several general advantages from various perspectives, as follows: (*i*) In the literature correlations, the critical properties (and sometimes acentric factors) were used as input parameters, whose calculations require indirect calculations as they often cannot be measured experimentally (by first calculating these properties for the HBA and HBD components separately, and then using a mixing rule to calculate the property for the DES). This makes the calculation of the input parameters difficult and time-consuming, while the method presented here requires no input parameters other than the groups presented in the tables, so the calculations are quite easy and fast; (*ii*) Furthermore, the models used for critical property and acentric factor calculations were developed for ionic liquids, not DESs, possibly resulting in high errors for these input properties when extended to DESs; (*iii*) A further issue is the comparison of the theoretical background of the models. The DES literature correlations are purely empirical in nature, and although they were developed for a large data bank on DESs, they are still merely empirical models. It is possible that their extrapolation to the new DESs of the future will produce high errors. However, the proposed GC/AC models are group/atomic contribution models, and in being so, they have a more solid theoretical background with respect to the purely empirical models. This is because the effects of the interactions of the various functional groups have been trained in the model development process, and therefore, they have more predictive characteristics; (*iv*) While the GC and AC models are both quite simple and their calculations are straightforward, the AC models in particular, are so simple that they can very easily be programmed and incorporated into software in a very straightforward manner. This is of great value in today’s academic and engineering world to have models which can be easily integrated into various software; (*v*) One further great advantage of the models of this work, similar to all other group contributions, is their independence of any experimental measurements on the DES. This easily allows for screening tests of DESs without actually requiring the DES to be prepared in laboratories, eliminating cost and time. This is invaluable in a field of science which is still at the infant stage, with innumerable numbers of DESs that can be envisioned.

While the above lists the advantages of the proposed models with respect to correlative approaches, it should be reminded that thermodynamic-based models can also be employed for the estimation of physical properties. However, since DESs are very complex mixtures involving hydrogen bonds, only the more elaborate and sophisticated thermodynamic models can handle such systems, so for example, the popular equations of state such as Peng-Robinson and Soave–Redlich–Kwong will render useless for DESs. Regardless, even the more thermodynamically suitable models, which are much-more cumbersome and time-consuming, are still not accurate if used in a purely theoretical (predictive) mode. Such thermodynamic models, for example the association-type equations of state, are fit to experimental data by the use of adjustable parameters which assist to reduce the errors. In this quest, while the thermodynamic models do indeed have higher predictability and extrapolative power as compared to the models presented here, this comes at the cost of losing the advantages mentioned in the previous paragraph for the proposed AC/GC models.

One further point of thought on the approach to take for physical property estimations of DESs, is the nature of DESs. In contrast to most solvents, which are pure, DESs are mixtures. Not only are they mixtures, but they are quite complex mixtures with various types if intermolecular interactions, including hydrogen bonds. This causes certain issues when attempting to model them, among which, is the choice to consider the DES as a pseudo-component or as a true mixture of two or more components. In many of the estimation models, such as global correlations and equations of state, input parameters such as the critical properties and acentric factors of the DES are required, which usually cannot be measured experimentally. If the pseudo-component approach is taken to estimate the values, the only procedure up to now, is to calculate the desired properties of the HBA and HBD components separately, followed by the use of a mixing rule to obtain, for example, the desired critical properties of the DES. This is not an ideal procedure, because the errors of the various steps build up, especially by considering the very nonideal behavior of the components in such a complex system. Unfortunately, there are still no such models available in literature. Therefore, the most serious challenge facing the pseudo-component pathway is to develop accurate models which can directly estimate the critical properties of the DES, or any other required input parameter for that matter. However, before such models become available, we suggest to avoid using correlations and semi-empirical models which use the critical properties of the DESs as their input parameters. Direct calculation models, such as the group/atomic contribution models are more suitable in this respect. Also, other models which would use only those physical properties of DESs which are experimentally measurable (such as molecular weight, density, viscosity, etc.) as their input parameters are suggested for higher accuracy. However, such methods can no longer be used for screening tests of novel envisioned DESs, while the GC/AC models can. On the other hand, the mindset of considering the DES as a true mixture of components, instead of one pseudo-component, also has its pros and cons. Such an approach is more theoretically realistic and it would be safer to use when extrapolations are called for. However, only very highly sophisticated thermodynamic approaches can handle the highly nonideal behavior of DES mixtures, i.e. detailed models that can see all the various types of physical phenomena and interactions in the hydrogen-bond networks. Furthermore, such models most often involve fitting parameters that must be optimized to experimental data. This would also prevent the use of such approaches as screening tools on DESs which have not yet been made in the labs. Furthermore, since such approaches are cumbersome and time-consuming, they are not the typical and commonplace techniques used by the research and engineering communities, and so there is the real risk that oversimplified models will be used, perhaps without realizing the extent of the risks of errors. Therefore, at the end of the day, there is still no one superior approach available and the proper choice of estimation technique is ultimately case-specific depending on the task at hand, the type and amount of information available, and the goal of estimations (for example as a screening tool). Due to all of the shortcomings and issues mentioned above, there is still much room for progress in this field and many challenges need to be overcome. However, due to exactly the variety of goals of the different users, it is urgent that all the different pathways be pursued and developed further, be it the simple engineering correlations based on physical property input, the group contribution approach which requires absolutely no physical property data, or the more elaborate approaches based on strong thermodynamic theories, such as equations of state, computational techniques, etc. Every single one of these pathways is still at its early stages for DESs and there is much room for progress in all. However, a serious obstacle in progress is the inevitable fact that DESs are only a newly-introduced category of solvents, hence, the amount of published physical property data is still insignificant compared to the number of potential DESs. This is even more serious for some of the less-investigated properties, such as speed of sound and heat capacity. The progress and accuracy of the modelling approaches go hand-in-hand with the extent and diversity of the physical property databanks. Therefore, parallel to researchers enriching the models, experimentalists need to contribute their share for true progress in the field.

## Methods

The basic procedure in group contribution models is that the molecular structure of a compound is considered to be made up of a number of functional groups. Specific numeric values, known as contributions or weights, are determined for each of the groups. The contribution of each of the groups is multiplied by the number of occurrences of that group in the structure, and the resulting summation on all the groups is considered within a mathematical function specific to the desired property. This procedure is highly dependent on how the chemical structure is decomposed. For complicated compounds, decomposition is not always easy. In some group contribution methods, it is even possible that the decomposition of the structure can be carried out in more than one way, with differing functional groups, and thus resulting in different calculated values for a property. In addition to this, structural decomposition into groups is a decisive task which is not easily programmable in computer software. While still following the mindset of the group contribution approach, atomic contribution models alleviate both of these issues. This is because in the AC procedure, the molecule is decomposed down to its atoms. Since the type and number of occurrences of these atoms are the only input parameters of the model, there is no risk of multiple methods of decomposition, and also, the simple approach makes it quite easily programmable and software-friendly. However, while AC models are very simple, they have absolutely no way of distinguishing the position of the atoms on the structure, and so they cannot differentiate isomers, or even different compounds with the same molecular formula.

By considering the specific advantages and disadvantages of each of the GC and AC models, we decided to propose both models for the estimation of densities, refractive indices, heat capacities, speeds of sound and surface tensions of DESs.

**Figure 4:**
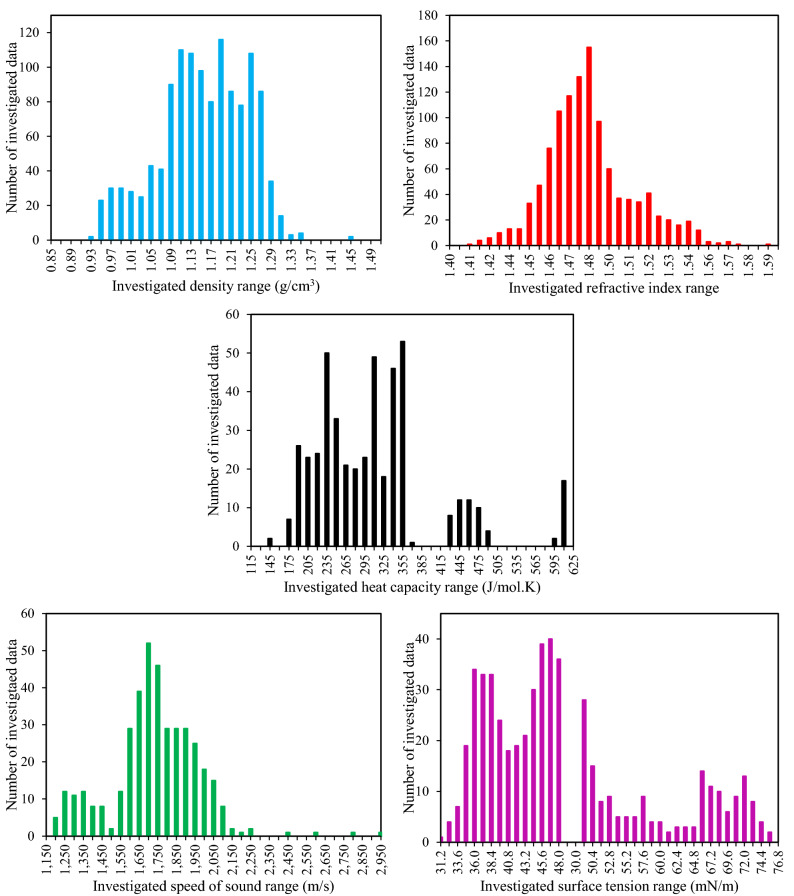
The ranges of investigated densities, refractive indices, heat capacities, speeds of sound and surface tensions and the corresponding data distributions.

In order to develop the GC and AC models, the most up-to-date databanks of various types of DESs were collected from the literature. The databanks involved 1239 data points from 149 DESs for density^35–66^, 1117 data points from 142 DESs for refractive index^11,39,41–43,47,50,54,57,59,63,64,67–87^, 461 data points from 24 DESs for heat capacity^59,70,88–93^, 398 data points from 37 DESs for speed of sound^42,43,67,72,75,77,82,94–100^ and 538 data points from 98 DESs for surface tension^47,63,71,80,86,87,101–116^. All of the data were at atmospheric pressure. Tables S1–S5 of the Supporting Information indicate the investigated DESs and the corresponding HBAs, HBDs, molar HBA/HBD ratios, molecular weights, and the number of data for density, refractive index, heat capacity, speed of sound, and surface tension, respectively. Furthermore, Fig. 4 illustrates the quantitative ranges of each of these properties, as well as the corresponding abundance of each in the databank.

The data collected on each physical property were divided randomly into the two groups of training (70–80%) and testing (20–30%). In this manner, in order to check the predictive ability of the models, a number of DESs were totally set aside and not used for development of the mathematical functionalities and the adjustable coefficients.

Various mathematical functionalities were investigated. For the sake of higher accuracy, functional groups or atoms were considered separately for the HBA and HBD structures. The GC and AC models were developed and optimized for each physical property with the aid of genetic algorithm. Equation 42 gives the objective function considered and applied to the training dataset.

42 $$ OF = \sum\limits_{i = 1}^{N} {\left| {\frac{{X_{i}^{Model} - X_{i}^{Exp} }}{{X_{i}^{Exp} }}} \right|} $$

where $X_{i}^{Model}$ is the calculated physical property by the GC or AC model and $X_{i}^{Exp}$ is the corresponding experimental value.

## Supplementary Information

Supplementary Information

## Data Availability

The data that support the findings of this work are available from the corresponding author upon reasonable request.
