# Supplementary material for: Group contribution and atomic contribution models for the prediction of various physical properties of deep eutectic solvents
Source: Sci Rep. 2021 Mar 23;11:6684. doi: 10.1038/s41598-021-85824-z (PMC7988013; doi:10.1038/s41598-021-85824-z)
Supplement: Supplementary file 1 — Supplementary Information [file 41598_2021_85824_MOESM1_ESM.docx]

**Supplementary Information**

**Group contribution and atomic contribution models for the prediction of various physical properties of deep eutectic solvents**

Reza Haghbakhsh^1,2^, Sona Raeissi2^,^, Ana Rita C. Duarte^*^^[[1]](#footnote-1)^

^1^ LAQV, REQUIMTE, Departamento de Química da Faculdade de Ciências e Tecnologia, Universidade Nova de Lisboa, 2829-516 Caparica, Portugal.

^2^ School of Chemical and Petroleum Engineering, Shiraz University, Mollasadra Ave., Shiraz 71348-51154, Iran.

Table S1. The list of investigated DESs with the corresponding information for developing the density models, and the resulting values of *AARD%* and *maximum ARD%* from the AC and GC models for each individual DES.

| # | HBA | HBD | Molar Ratio HBA:HBD | Mw (g/mol) | No. of data points | AC results | | GC results | | Ref. |
| --- | --- | --- | --- | --- | --- | --- | --- | --- | --- | --- |
|  |  |  |  |  |  | AARD% | Max ARD% | AARD% | Max ARD% |  |
| 1 | Acetyl choline chloride | 1,2,4-triazole | 1:1 | 125.36 | 7 | 0.19 | 0.37 | 0.72 | 1.50 | 35 |
| 2 | Acetyl choline chloride | D-Fructose | 1:1 | 180.91 | 13 | 0.68 | 1.55 | 1.03 | 1.53 | 36 |
| 3 | Acetyl choline chloride | D-Glucose | 1:1 | 180.91 | 13 | 0.61 | 1.30 | 0.65 | 0.96 | 36 |
| 4 | Acetyl choline chloride | D-Mannose | 1:1 | 180.91 | 13 | 0.70 | 1.58 | 0.15 | 0.27 | 36 |
| 5 | Acetyl choline chloride | D-Ribose | 1:1 | 165.90 | 13 | 0.66 | 1.39 | 0.25 | 0.51 | 36 |
| 6 | Acetyl choline chloride | D-Xylose | 1:1 | 165.90 | 13 | 0.62 | 1.31 | 0.21 | 0.43 | 36 |
| 7 | Acetyl choline chloride | Guaiacol | 1:3 | 138.52 | 4 | 1.61 | 1.85 | 0.31 | 0.57 | 37 |
| 8 | Acetyl choline chloride | Guaiacol | 1:4 | 135.64 | 4 | 2.34 | 2.59 | 0.32 | 0.56 | 37 |
| 9 | Acetyl choline chloride | Guaiacol | 1:5 | 133.73 | 4 | 2.93 | 3.08 | 0.30 | 0.47 | 37 |
| 10 | Acetyl choline chloride | Imidazole | 1:1.5 | 113.51 | 7 | 0.19 | 0.30 | 0.43 | 0.92 | 38 |
| 11 | Acetyl choline chloride | Imidazole | 1:2 | 105.94 | 7 | 3.00 | 3.19 | 0.93 | 1.35 | 38 |
| 12 | Acetyl choline chloride | Imidazole | 1:3 | 96.47 | 7 | 6.86 | 8.09 | 0.76 | 0.98 | 38 |
| 13 | Acetyl choline chloride | Levulinic acid | 1:1 | 148.89 | 11 | 6.41 | 7.83 | 1.48 | 2.87 | 39 |
| 14 | Acetyl choline chloride | Levulinic acid | 1:3 | 132.50 | 4 | 2.54 | 3.19 | 0.53 | 1.08 | 40 |
| 15 | Allyl triphenylphosphonium bromide | Diethylene glycol | 1:4 | 161.54 | 11 | 3.55 | 4.68 | 0.71 | 1.61 | 41 |
| 16 | Allyl triphenylphosphonium bromide | Diethylene glycol | 1:10 | 131.31 | 11 | 1.12 | 1.91 | 0.49 | 0.91 | 41 |
| 17 | Allyl triphenylphosphonium bromide | Diethylene glycol | 1:16 | 122.42 | 11 | 1.68 | 2.19 | 2.09 | 2.73 | 41 |
| 18 | Allyl triphenylphosphonium bromide | Triethylene glycol | 1:4 | 196.79 | 11 | 1.30 | 2.26 | 0.68 | 1.31 | 41 |
| 19 | Allyl triphenylphosphonium bromide | Triethylene glycol | 1:10 | 171.36 | 11 | 3.29 | 3.78 | 1.88 | 2.79 | 41 |
| 20 | Allyl triphenylphosphonium bromide | Triethylene glycol | 1:16 | 163.88 | 11 | 0.10 | 0.33 | 0.41 | 0.82 | 41 |
| 21 | Benzyl tripropyl ammonium Chloride | Ethylene Glycol | 1:3 | 114.02 | 11 | 0.78 | 1.66 | 0.68 | 1.46 | 42 |
| 22 | Benzyl tripropyl ammonium Chloride | Glycerol | 1:3 | 136.53 | 11 | 4.31 | 5.14 | 3.32 | 4.42 | 42 |
| 23 | Benzyl tripropyl ammonium Chloride | Lactic Acid | 1:3 | 135.02 | 11 | 0.66 | 1.44 | 1.92 | 3.18 | 42 |
| 24 | Benzyl tripropyl ammonium Chloride | Oxalic acid | 1:1 | 179.95 | 12 | 6.37 | 7.52 | 9.73 | 10.93 | 42 |
| 25 | Benzyl tripropyl ammonium Chloride | Phenol | 1:3 | 138.05 | 11 | 0.58 | 1.08 | 0.70 | 1.59 | 42 |
| 26 | Betaine | Lactic acid | 1:2 | 99.10 | 10 | 0.68 | 1.44 | 0.88 | 1.89 | 43 |
| 27 | Betaine | Lactic acid | 1:5 | 94.59 | 11 | 0.66 | 1.44 | 1.09 | 2.34 | 43 |
| 28 | Betaine | Levulinic acid | 1:2 | 116.46 | 11 | 1.39 | 2.48 | 1.41 | 2.38 | 43 |
| 29 | Benzyl dimethyl(2-hydroxyethyl) ammonium chloride | D-Fructose | 1:1 | 197.94 | 13 | 0.65 | 1.42 | 1.79 | 2.40 | 36 |
| 30 | Benzyl dimethyl(2-hydroxyethyl) ammonium chloride | D-Glucose | 1:1 | 197.94 | 13 | 1.07 | 2.04 | 0.26 | 0.66 | 36 |
| 31 | Benzyl dimethyl(2-hydroxyethyl) ammonium chloride | D-Mannose | 1:1 | 197.94 | 13 | 1.70 | 2.59 | 0.37 | 0.57 | 36 |
| 32 | Benzyl dimethyl(2-hydroxyethyl) ammonium chloride | D-Ribose | 1:1 | 182.93 | 13 | 1.35 | 2.42 | 0.32 | 0.63 | 36 |
| 33 | Benzyl dimethyl(2-hydroxyethyl) ammonium chloride | D-Xylose | 1:1 | 182.93 | 13 | 0.57 | 1.21 | 1.30 | 1.80 | 36 |
| 34 | Choline chloride | 1,2-propanediol | 1:3 | 91.98 | 4 | 1.38 | 1.99 | 1.01 | 1.86 | 44 |
| 35 | Choline chloride | 1,2-propanediol | 1:4 | 88.80 | 4 | 0.31 | 0.60 | 0.74 | 1.47 | 44 |
| 36 | Choline chloride | 1,4-butanediol | 1:3 | 102.50 | 4 | 0.28 | 0.64 | 0.35 | 0.62 | 44 |
| 37 | Choline chloride | 1,4-butanediol | 1:4 | 100.02 | 5 | 1.14 | 1.39 | 0.85 | 1.20 | 44,45 |
| 38 | Choline chloride | 2,3-butanediol | 1:3 | 102.50 | 4 | 1.00 | 1.46 | 0.68 | 1.26 | 44 |
| 39 | Choline chloride | 2,3-butanediol | 1:4 | 100.02 | 4 | 0.31 | 0.48 | 1.22 | 1.88 | 44 |
| 40 | Choline chloride | Acetamide | 1:2 | 85.92 | 1 | 2.76 | 2.76 | 3.80 | 3.80 | 46 |
| 41 | Choline chloride | Citric acid | 1:1 | 165.87 | 2 | 1.28 | 2.51 | 11.57 | 12.93 | 45,46 |
| 42 | Choline chloride | D-Fructose | 1:1 | 159.89 | 7 | 6.78 | 7.56 | 4.24 | 4.30 | 47 |
| 43 | Choline chloride | D-Fructose | 1.5:1 | 155.83 | 7 | 9.62 | 10.25 | 2.66 | 2.73 | 47 |
| 44 | Choline chloride | D-Fructose | 2:1 | 153.13 | 7 | 10.50 | 11.03 | 1.29 | 1.37 | 47 |
| 45 | Choline chloride | D-Fructose | 2.5:1 | 151.20 | 7 | 10.66 | 10.96 | 0.34 | 0.47 | 47 |
| 46 | Choline chloride | D-Glucose | 1:1 | 159.89 | 1 | 2.65 | 2.65 | 0.63 | 0.63 | 46 |
| 47 | Choline chloride | D-Glucose | 2:1 | 153.13 | 6 | 8.04 | 8.42 | 1.18 | 1.36 | 48 |
| 48 | Choline chloride | D-Mannose | 1:1 | 159.89 | 13 | 2.59 | 3.44 | 0.13 | 0.19 | 36 |
| 49 | Choline chloride | D-Ribose | 1:1 | 144.88 | 13 | 2.92 | 3.80 | 0.66 | 0.84 | 36 |
| 50 | Choline chloride | D-Sorbitol | 1:1 | 159.89 | 1 | 4.75 | 4.75 | 4.31 | 4.31 | 45 |
| 51 | Choline chloride | D-Sucrose | 1:1 | 240.96 | 1 | 3.53 | 3.53 | 1.44 | 1.44 | 46 |
| 52 | Choline chloride | D-Sucrose | 2:1 | 240.96 | 1 | 6.54 | 6.54 | 19.28 | 19.28 | 46 |
| 53 | Choline chloride | D-Sucrose | 4:1 | 180.16 | 1 | 7.34 | 7.34 | 12.54 | 12.54 | 46 |
| 54 | Choline chloride | D-Xylose | 1:1 | 144.88 | 13 | 2.23 | 3.11 | 0.08 | 0.23 | 36 |
| 55 | Choline chloride | D-Xylose | 3:1 | 142.25 | 1 | 8.78 | 8.78 | 0.01 | 0.01 | 46 |
| 56 | Choline chloride | Ethylene glycol | 1:1.78 | 89.96 | 15 | 1.42 | 2.69 | 1.08 | 2.52 | 49 |
| 57 | Choline chloride | Ethylene glycol | 1:2 | 87.92 | 30 | 0.92 | 2.59 | 0.79 | 2.52 | 50-52 |
| 58 | Choline chloride | Ethylene glycol | 1:2.03 | 87.66 | 15 | 1.01 | 2.23 | 0.94 | 2.22 | 49 |
| 59 | Choline chloride | Ethylene glycol | 1:2.57 | 83.79 | 15 | 0.62 | 1.42 | 0.82 | 1.70 | 49 |
| 60 | Choline chloride | Glutaric acid | 1:1 | 135.87 | 13 | 2.52 | 3.67 | 1.55 | 2.80 | 53 |
| 61 | Choline chloride | Glycerol | 1:1 | 115.86 | 15 | 2.01 | 3.20 | 2.50 | 4.03 | 49 |
| 62 | Choline chloride | Glycerol | 1:2 | 107.94 | 18 | 3.31 | 4.29 | 0.99 | 2.18 | 45,50,53 |
| 63 | Choline chloride | Glycerol | 1:2.03 | 107.78 | 15 | 3.15 | 4.01 | 0.76 | 1.60 | 49 |
| 64 | Choline chloride | Glycerol | 1:3 | 103.98 | 15 | 4.97 | 5.69 | 0.77 | 1.63 | 49 |
| 65 | Choline chloride | Glycolic acid | 1:1 | 107.84 | 13 | 0.78 | 1.69 | 1.22 | 2.42 | 54 |
| 66 | Choline chloride | Guaiacol | 1:3 | 128.01 | 4 | 2.61 | 3.00 | 0.66 | 1.24 | 37 |
| 67 | Choline chloride | Guaiacol | 1:4 | 127.24 | 4 | 3.27 | 3.78 | 0.85 | 1.57 | 37 |
| 68 | Choline chloride | Guaiacol | 1:5 | 126.72 | 4 | 3.04 | 3.38 | 0.52 | 0.95 | 37 |
| 69 | Choline chloride | Levulinic acid | 1:1 | 127.87 | 11 | 4.52 | 5.79 | 0.80 | 1.81 | 39 |
| 70 | Choline chloride | Levulinic acid | 1:2 | 123.95 | 13 | 2.73 | 3.88 | 0.75 | 1.61 | 45,54 |
| 71 | Choline chloride | Levulinic acid | 1:3 | 121.99 | 4 | 1.84 | 2.40 | 0.41 | 0.80 | 55 |
| 72 | Choline chloride | Levulinic acid | 1:4 | 120.81 | 4 | 1.59 | 2.10 | 0.91 | 1.37 | 55 |
| 73 | Choline chloride | Levulinic acid | 1:5 | 120.03 | 4 | 1.72 | 2.15 | 2.60 | 2.93 | 55 |
| 74 | Choline chloride | Malonic acid | 1:1 | 121.84 | 13 | 0.96 | 1.78 | 2.41 | 3.57 | 51, 54 |
| 75 | Choline chloride | Malonic acid | 1:2 | 115.91 | 9 | 7.83 | 9.40 | 3.67 | 5.59 | 51 |
| 76 | Choline chloride | furfuryl alcohol | 1:3 | 108.48 | 4 | 0.66 | 1.15 | 0.45 | 0.72 | 55 |
| 77 | Choline chloride | furfuryl alcohol | 1:4 | 106.40 | 4 | 0.58 | 1.07 | 0.46 | 0.80 | 55 |
| 78 | Choline chloride | furfuryl alcohol | 1:5 | 105.02 | 4 | 0.86 | 1.27 | 0.42 | 0.69 | 55 |
| 79 | Choline chloride | O-Cresol | 1:2 | 118.63 | 1 | 1.54 | 1.54 | 1.47 | 1.47 | 56 |
| 80 | Choline chloride | O-Cresol | 1:3 | 116.01 | 1 | 0.01 | 0.01 | 0.83 | 0.83 | 56 |
| 81 | Choline chloride | O-Cresol | 1:3.91 | 114.55 | 1 | 0.79 | 0.79 | 0.71 | 0.71 | 56 |
| 82 | Choline chloride | O-Cresol | 1:5 | 113.39 | 1 | 1.25 | 1.25 | 0.91 | 0.91 | 56 |
| 83 | Choline chloride | O-Cresol | 1:6 | 112.64 | 1 | 1.29 | 1.29 | 1.40 | 1.40 | 56 |
| 84 | Choline chloride | Oxalic acid | 1:1 | 114.83 | 1 | 0.81 | 0.81 | 2.09 | 2.09 | 45,54 |
| 85 | Choline chloride | P-Chlorophenol | 1:2 | 132.24 | 9 | 0.54 | 1.24 | 0.68 | 1.24 | 57 |
| 86 | Choline chloride | P-Cresol | 1:2 | 118.63 | 9 | 3.33 | 4.52 | 3.38 | 4.73 | 57 |
| 87 | Choline chloride | Phenol | 1:2 | 109.28 | 6 | 1.46 | 1.88 | 0.42 | 0.89 | 56 |
| 88 | Choline chloride | Phenol | 1:3 | 105.49 | 6 | 0.31 | 0.69 | 0.38 | 0.78 | 56 |
| 89 | Choline chloride | Phenol | 1:4 | 103.21 | 6 | 0.21 | 0.41 | 0.42 | 0.83 | 56 |
| 90 | Choline chloride | Phenol | 1:5 | 101.70 | 6 | 0.21 | 0.36 | 0.29 | 0.53 | 56 |
| 91 | Choline chloride | Phenol | 1:6 | 100.61 | 6 | 0.24 | 0.48 | 0.42 | 0.81 | 56 |
| 92 | Choline chloride | p-Toluenesulfonic acid | 1:1 | 158.38 | 1 | 1.04 | 1.04 | 0.12 | 0.12 | 45 |
| 93 | Choline chloride | Tartaric acid | 1:1 | 144.85 | 1 | 1.79 | 1.79 | 4.98 | 4.98 | 46 |
| 94 | Choline chloride | Tartaric acid | 2:1 | 143.11 | 1 | 8.90 | 8.90 | 9.94 | 9.94 | 45 |
| 95 | Choline chloride | Triethylene glycol | 1:2 | 146.65 | 6 | 1.52 | 2.61 | 1.63 | 2.92 | 48 |
| 96 | Choline chloride | Triethylene glycol | 1:4 | 148.06 | 1 | 2.65 | 2.65 | 1.69 | 1.69 | 45 |
| 97 | Choline chloride | Urea | 1:2 | 86.58 | 21 | 0.79 | 1.38 | 0.58 | 1.95 | 45,58-61 |
| 98 | Choline chloride | Xylitol | 1:1 | 145.88 | 1 | 3.31 | 3.31 | 1.90 | 1.90 | 45 |
| 99 | Citric acid | D-Glucose | 1:1 | 186.14 | 1 | 34.35 | 34.35 | 2.52 | 2.52 | 46 |
| 100 | D-Glucose | Tartaric acid | 1:1 | 165.12 | 1 | 42.80 | 42.80 | 5.05 | 5.05 | 46 |
| 101 | Diethylamine hydrochloride | Guaiacol | 1:3 | 120.50 | 4 | 1.80 | 2.45 | 0.53 | 0.87 | 37 |
| 102 | Diethylamine hydrochloride | Guaiacol | 1:4 | 121.23 | 4 | 0.66 | 1.07 | 0.44 | 0.74 | 37 |
| 103 | Diethylamine hydrochloride | Guaiacol | 1:5 | 121.72 | 4 | 0.38 | 0.98 | 0.53 | 0.90 | 37 |
| 104 | L-proline | Lactic acid | 1:1 | 102.61 | 11 | 3.52 | 4.43 | 0.66 | 1.51 | 43 |
| 105 | L-proline | Levulinic acid | 1:2 | 115.78 | 11 | 0.74 | 1.63 | 0.63 | 1.37 | 43 |
| 106 | Methyl triphenylphosphonium bromide | Ethylene glycol | 1:3 | 135.86 | 15 | 0.84 | 2.07 | 1.25 | 2.47 | 49 |
| 107 | Methyl triphenylphosphonium bromide | Ethylene glycol | 1:4 | 121.10 | 22 | 0.67 | 1.37 | 0.73 | 1.55 | 49, 62 |
| 108 | Methyl triphenylphosphonium bromide | Ethylene glycol | 1:5.25 | 109.29 | 15 | 1.00 | 1.96 | 0.70 | 1.72 | 49 |
| 109 | Methyl triphenylphosphonium bromide | Glycerol | 1:1.75 | 188.50 | 7 | 3.80 | 5.10 | 0.87 | 1.93 | 62 |
| 110 | Methyl triphenylphosphonium bromide | Glycerol | 1:2.03 | 179.59 | 15 | 1.24 | 2.60 | 0.87 | 2.00 | 49 |
| 111 | Methyl triphenylphosphonium bromide | Glycerol | 1:3 | 158.38 | 15 | 1.69 | 2.84 | 0.75 | 1.52 | 49 |
| 112 | Methyl triphenylphosphonium bromide | Glycerol | 1:4 | 145.12 | 15 | 3.19 | 4.23 | 1.31 | 2.76 | 49 |
| 113 | N,N-diethylenethanolammonium chloride | Ethylene glycol | 1:2 | 92.60 | 10 | 1.38 | 2.21 | 0.76 | 1.72 | 52 |
| 114 | N,N-diethylenethanolammonium chloride | Ethylene glycol | 1:2.03 | 92.29 | 15 | 1.89 | 3.33 | 1.40 | 3.19 | 49 |
| 115 | N,N-diethylenethanolammonium chloride | Ethylene glycol | 1:3 | 84.96 | 15 | 0.73 | 1.98 | 1.02 | 2.07 | 49 |
| 116 | N,N-diethylenethanolammonium chloride | Ethylene glycol | 1:4 | 80.38 | 15 | 0.72 | 1.54 | 1.32 | 2.91 | 49 |
| 117 | N,N-diethylenethanolammonium chloride | Glycerol | 1:2 | 112.61 | 10 | 2.87 | 3.60 | 0.67 | 1.47 | 52 |
| 118 | N,N-diethylenethanolammonium chloride | Glycerol | 1:2.03 | 112.41 | 15 | 2.23 | 3.35 | 1.35 | 3.07 | 49 |
| 119 | N,N-diethylenethanolammonium chloride | Glycerol | 1:3 | 107.48 | 15 | 5.80 | 6.73 | 1.05 | 2.41 | 49 |
| 120 | N,N-diethylenethanolammonium chloride | Glycerol | 1:4 | 104.41 | 15 | 7.46 | 8.19 | 1.12 | 2.52 | 49 |
| 121 | Tetrabutylammonium chloride | Arginine | 6:1 | 263.10 | 6 | 1.56 | 2.01 | 0.99 | 1.73 | 41 |
| 122 | Tetrabutylammonium chloride | Arginine | 7:1 | 264.95 | 6 | 0.38 | 0.73 | 0.59 | 1.29 | 41 |
| 123 | Tetrabutylammonium chloride | Arginine | 8:1 | 266.39 | 6 | 0.26 | 0.47 | 0.40 | 0.75 | 41 |
| 124 | Tetrabutylammonium chloride | Aspartic acid | 9:1 | 263.44 | 6 | 0.30 | 0.55 | 0.43 | 0.82 | 41 |
| 125 | Tetrabutylammonium chloride | Aspartic acid | 10:1 | 264.75 | 6 | 0.78 | 1.28 | 0.68 | 1.40 | 41 |
| 126 | Tetrabutylammonium chloride | Aspartic acid | 11:1 | 265.85 | 6 | 1.59 | 1.97 | 1.40 | 2.02 | 41 |
| 127 | Tetrabutylammonium chloride | Ethylene glycol | 1:2 | 96.61 | 7 | 9.59 | 10.75 | 9.10 | 10.33 | 63 |
| 128 | Tetrabutylammonium chloride | Ethylene glycol | 1:3 | 127.14 | 7 | 8.82 | 10.07 | 8.81 | 10.12 | 63 |
| 129 | Tetrabutylammonium chloride | Ethylene glycol | 1:4 | 82.80 | 7 | 13.47 | 14.36 | 12.15 | 13.17 | 63 |
| 130 | Tetrabutylammonium chloride | Glutamic acid | 8:1 | 263.39 | 6 | 0.78 | 1.36 | 0.72 | 1.43 | 41 |
| 131 | Tetrabutylammonium chloride | Glutamic acid | 9:1 | 264.84 | 6 | 0.43 | 0.81 | 0.51 | 0.93 | 41 |
| 132 | Tetrabutylammonium chloride | Glutamic acid | 10:1 | 266.03 | 6 | 0.42 | 0.92 | 0.51 | 1.16 | 41 |
| 133 | Tetrabutylammonium chloride | Glycerol | 1:3 | 138.55 | 7 | 3.85 | 5.39 | 3.85 | 5.60 | 63 |
| 134 | Tetrabutylammonium chloride | Glycerol | 1:4 | 129.26 | 7 | 1.73 | 3.11 | 2.17 | 3.85 | 63 |
| 135 | Tetrabutylammonium chloride | Glycerol | 1:5 | 123.06 | 7 | 0.90 | 2.04 | 1.40 | 3.05 | 63 |
| 136 | Tetrabutylammonium chloride | Phenylacetic acid | 1:2 | 146.00 | 11 | 0.64 | 1.30 | 0.66 | 1.37 | 64 |
| 137 | Tetrabutylammonium chloride | Propionic acid | 1:2 | 142.03 | 11 | 8.13 | 9.69 | 6.23 | 7.78 | 64 |
| 138 | Tetrabutylammonium chloride | Triethylene glycol | 1:1 | 214.04 | 7 | 8.36 | 10.11 | 5.32 | 7.05 | 63 |
| 139 | Tetrabutylammonium chloride | Triethylene glycol | 2:1 | 235.33 | 7 | 1.50 | 3.12 | 1.14 | 2.57 | 63 |
| 140 | Tetrabutylammonium chloride | Triethylene glycol | 3:1 | 245.98 | 7 | 1.64 | 2.99 | 1.24 | 2.57 | 63 |
| 141 | Tetrabutylammonium chloride | Triethylene glycol | 4:1 | 252.37 | 7 | 3.42 | 4.52 | 2.38 | 3.57 | 63 |
| 142 | Tetraethylammonium bromide | Levulinic acid | 1:1 | 163.14 | 11 | 3.26 | 4.35 | 3.89 | 4.90 | 39 |
| 143 | Tetraethylammonium chloride | Levulinic acid | 1:1 | 140.91 | 11 | 4.34 | 5.75 | 0.79 | 1.76 | 39 |
| 144 | Tetrahexylammonium bromide | Ethylene glycol | 1:2 | 186.24 | 11 | 6.34 | 7.93 | 3.18 | 4.59 | 65 |
| 145 | Tetrahexylammonium bromide | Glycerol | 1:2 | 206.26 | 11 | 4.49 | 5.95 | 0.99 | 2.20 | 65 |
| 146 | Trimethylglycine | 2-Chloro benzoic acid | 1:2 | 143.43 | 8 | 2.06 | 4.52 | 1.36 | 2.62 | 66 |
| 147 | Trimethylglycine | Benzoic acid | 1:2 | 120.46 | 3 | 1.68 | 2.64 | 1.42 | 2.42 | 66 |
| 148 | Trimethylglycine | Mandelic acid | 1:2 | 140.48 | 5 | 2.14 | 3.08 | 0.85 | 1.62 | 66 |
| 149 | Trimethylglycine | Phenylacetic acid | 1:2 | 129.82 | 5 | 1.16 | 1.69 | 1.17 | 2.03 | 66 |

Table S2. The list of investigated DESs with the corresponding information for developing the refractive index models, and the resulting values of *AARD%* and *maximum ARD%* from the AC and GC models for each individual DES.

| # | HBA | HBD | Molar Ratio HBA:HBD | Mw (g/mol) | No. of data points | AC results | | GC results | | Ref. |
| --- | --- | --- | --- | --- | --- | --- | --- | --- | --- | --- |
|  |  |  |  |  |  | AARD% | Max ARD% | AARD% | Max ARD% |  |
| 1 | Acetylcholine chloride | Levulinic acid | 1:1 | 148.89 | 11 | 2.17 | 2.64 | 0.77 | 0.95 | 39 |
| 2 | Allyl triphenyl phosphonium bromide | Diethylene glycol | 1:4 | 161.54 | 11 | 3.05 | 3.29 | 0.14 | 0.25 | 41 |
| 3 | Allyl triphenyl phosphonium bromide | Diethylene glycol | 1:10 | 131.31 | 11 | 0.64 | 0.93 | 0.14 | 0.32 | 41 |
| 4 | Allyl triphenyl phosphonium bromide | Diethylene glycol | 1:16 | 122.42 | 11 | 0.17 | 0.41 | 0.13 | 0.28 | 41 |
| 5 | Allyl triphenyl phosphonium bromide | Triethylene glycol | 1:4 | 196.79 | 11 | 2.17 | 2.32 | 0.14 | 0.32 | 41 |
| 6 | Allyl triphenyl phosphonium bromide | Triethylene glycol | 1:10 | 171.36 | 11 | 0.10 | 0.17 | 0.16 | 0.33 | 41 |
| 7 | Allyl triphenyl phosphonium bromide | Triethylene glycol | 1:16 | 163.88 | 11 | 1.05 | 1.16 | 0.13 | 0.33 | 41 |
| 8 | Benzyl tributylammonium chloride | Diethylene glycol | 1:3 | 157.57 | 13 | 0.88 | 1.32 | 0.24 | 0.52 | 67 |
| 9 | Benzyl tributylammonium chloride | Ethylene glycol | 1:3 | 124.53 | 13 | 1.02 | 1.53 | 0.32 | 0.63 | 67 |
| 10 | Benzyl tributylammonium chloride | Glycerol | 1:3 | 147.05 | 13 | 1.77 | 2.20 | 0.31 | 0.50 | 67 |
| 11 | Benzyl tributylammonium chloride | Triethylene glycol | 1:3 | 190.61 | 13 | 0.68 | 1.05 | 0.23 | 0.49 | 67 |
| 12 | Benzyl triethylammonium chloride | Citric acid | 1:1 | 209.95 | 9 | 1.97 | 2.14 | 0.52 | 0.70 | 68 |
| 13 | Benzyl triethylammonium chloride | Oxalic acid | 1:1 | 158.90 | 9 | 0.91 | 1.21 | 0.44 | 0.71 | 68 |
| 14 | Benzyl triethylammonium chloride | P-toluene sulfonic acid | 3:7 | 189.42 | 9 | 1.43 | 2.36 | 0.11 | 0.17 | 68 |
| 15 | Benzyl trimethylammonium chloride | Citric acid | 1:1 | 188.91 | 9 | 1.36 | 1.55 | 0.18 | 0.39 | 69 |
| 16 | Benzyl trimethylammonium chloride | Diethylene glycol | 1:3 | 126.01 | 13 | 0.72 | 1.16 | 0.23 | 0.54 | 67 |
| 17 | Benzyl trimethylammonium chloride | Ethylene glycol | 1:3 | 92.97 | 13 | 0.64 | 1.16 | 0.22 | 0.53 | 67 |
| 18 | Benzyl trimethylammonium chloride | Glycerol | 1:3 | 115.49 | 13 | 1.82 | 2.19 | 0.09 | 0.15 | 67 |
| 19 | Benzyl trimethylammonium chloride | Oxalic acid | 1:1 | 137.86 | 9 | 0.19 | 0.44 | 0.20 | 0.44 | 69 |
| 20 | Benzyl trimethylammonium chloride | P-toluene sulfonic acid | 3:7 | 170.08 | 9 | 0.85 | 1.65 | 0.57 | 0.74 | 69 |
| 21 | Benzyl trimethylammonium chloride | Triethylene glycol | 1:3 | 159.05 | 13 | 0.59 | 0.98 | 0.25 | 0.58 | 67 |
| 22 | Benzyl tripropyl ammonium chloride | Ethylene glycol | 1:3 | 114.02 | 11 | 0.98 | 1.40 | 0.26 | 0.51 | 42 |
| 23 | Benzyl tripropyl ammonium chloride | Glycerol | 1:3 | 136.53 | 11 | 1.57 | 1.92 | 0.12 | 0.20 | 42 |
| 24 | Benzyl tripropyl ammonium chloride | Lactic acid | 1:3 | 135.02 | 11 | 0.32 | 0.70 | 0.27 | 0.56 | 42 |
| 25 | Benzyl tripropyl ammonium chloride | Phenol | 1:3 | 138.05 | 11 | 0.21 | 0.43 | 1.03 | 1.26 | 42 |
| 26 | Betaine | DL-lactic acid | 1:2 | 99.10 | 11 | 2.13 | 2.56 | 0.24 | 0.48 | 43 |
| 27 | Betaine | DL-lactic acid | 1:5 | 94.59 | 11 | 2.89 | 3.35 | 0.73 | 1.17 | 43 |
| 28 | Betaine | Ethylene glycol | 1:3 | 75.84 | 5 | 1.87 | 2.20 | 0.20 | 0.37 | 70 |
| 29 | Betaine | Glycerol | 1:3 | 98.36 | 5 | 0.21 | 0.39 | 0.91 | 1.19 | 70 |
| 30 | Betaine | Levulinic acid | 1:2 | 116.46 | 11 | 2.01 | 2.42 | 0.42 | 1.19 | 43 |
| 31 | Betaine | Propylene Glycol | 1:3.5 | 85.21 | 5 | 1.88 | 2.22 | 0.43 | 0.68 | 70 |
| 32 | Butylammonium bromide | Glycerol | 1:2 | 112.75 | 1 | 1.31 | 1.31 | 0.27 | 0.27 | 71 |
| 35 | Choline chloride | 1,2-propanediol | 1:3 | 91.98 | 10 | 1.15 | 1.58 | 0.75 | 1.07 | 72 |
| 33 | Choline Chloride | 1,4-butanediol | 1:2 | 106.62 | 1 | 0.14 | 0.14 | 0.01 | 0.01 | 73 |
| 34 | Choline Chloride | 1,4-butanediol | 1:2.57 | 103.99 | 1 | 0.29 | 0.29 | 0.03 | 0.03 | 73 |
| 36 | Choline Chloride | 1,4-butanediol | 1:3 | 102.50 | 1 | 0.38 | 0.38 | 0.04 | 0.04 | 73 |
| 37 | Choline chloride | D-fructose | 1:1 | 159.89 | 7 | 2.74 | 3.03 | 0.19 | 0.53 | 47 |
| 38 | Choline chloride | D-fructose | 1.5:1 | 155.83 | 7 | 2.71 | 3.04 | 0.19 | 0.45 | 47 |
| 39 | Choline chloride | D-fructose | 2:1 | 153.13 | 7 | 2.72 | 3.02 | 0.21 | 0.46 | 47 |
| 40 | Choline chloride | D-fructose | 2.5:1 | 151.20 | 7 | 2.97 | 3.29 | 0.31 | 0.60 | 47 |
| 41 | Choline Chloride | Ethylene glycol | 1:1.78 | 89.96 | 1 | 0.74 | 0.74 | 0.62 | 0.62 | 50 |
| 42 | Choline Chloride | Ethylene glycol | 1:2 | 87.92 | 9 | 0.96 | 1.27 | 0.77 | 1.04 | 50 |
| 43 | Choline Chloride | Ethylene glycol | 1:2.57 | 83.79 | 1 | 1.07 | 1.07 | 0.79 | 0.79 | 50 |
| 44 | Choline chloride | Glutaric acid | 1:1 | 135.87 | 13 | 0.32 | 0.73 | 0.25 | 0.49 | 74 |
| 45 | Choline Chloride | Glycerol | 1:1 | 115.86 | 1 | 0.85 | 0.85 | 0.32 | 0.32 | 73 |
| 46 | Choline Chloride | Glycerol | 1:2 | 107.94 | 40 | 0.50 | 0.89 | 0.16 | 0.42 | 50, 73,75,76 |
| 47 | Choline chloride | Glycerol | 1:3 | 103.98 | 8 | 0.38 | 0.65 | 0.19 | 0.39 | 76 |
| 48 | Choline chloride | Glycerol | 1:4 | 101.60 | 8 | 0.37 | 0.63 | 0.25 | 0.43 | 76 |
| 49 | Choline chloride | Glycolic acid | 1:1 | 107.84 | 13 | 0.44 | 0.90 | 0.33 | 0.74 | 54 |
| 50 | Choline chloride | Levulinic acid | 1:1 | 127.87 | 11 | 1.84 | 2.32 | 0.19 | 0.45 | 39 |
| 51 | Choline chloride | Levulinic acid | 1:2 | 123.95 | 23 | 1.33 | 1.83 | 0.11 | 0.22 | 54, 77 |
| 52 | Choline chloride | Malonic acid | 1:1 | 121.84 | 13 | 0.28 | 0.64 | 0.23 | 0.50 | 54,78 |
| 53 | Choline chloride | Mono chloroacetic acid | 1:2 | 109.54 | 11 | 1.10 | 1.48 | 0.21 | 0.45 | 79 |
| 54 | Choline chloride | Mono ethanolamine | 1:5 | 74.17 | 7 | 0.52 | 1.17 | 0.41 | 0.83 | 80 |
| 55 | Choline chloride | Mono ethanolamine | 1:6 | 72.30 | 7 | 0.49 | 1.10 | 0.31 | 0.63 | 80 |
| 56 | Choline chloride | Mono ethanolamine | 1:7 | 70.90 | 7 | 0.58 | 1.20 | 0.23 | 0.40 | 80 |
| 57 | Choline chloride | Mono ethanolamine | 1:8 | 69.81 | 7 | 0.62 | 1.22 | 0.19 | 0.44 | 80 |
| 58 | Choline chloride | Oxalic acid | 1:1 | 114.83 | 12 | 0.45 | 0.90 | 0.26 | 0.56 | 54 |
| 59 | Choline chloride | P-chlorophenol | 1:2 | 132.24 | 9 | 0.14 | 0.30 | 0.02 | 0.06 | 57 |
| 60 | Choline chloride | P-cresol | 1:2 | 118.63 | 9 | 1.52 | 1.74 | 0.74 | 0.76 | 57 |
| 61 | Choline chloride | Phenol | 1:2 | 109.28 | 9 | 1.17 | 1.41 | 0.02 | 0.03 | 57 |
| 62 | Choline chloride | Propionic acid | 1:2 | 95.93 | 11 | 2.50 | 2.80 | 1.15 | 1.45 | 79 |
| 63 | Choline chloride | P-toluene sulfonic acid | 1:2 | 161.34 | 11 | 1.41 | 1.46 | 0.40 | 0.71 | 79 |
| 64 | Choline chloride | Trichloroacetic acid | 1:2 | 155.47 | 11 | 0.42 | 0.86 | 0.15 | 0.31 | 79 |
| 65 | Choline chloride | Urea | 1:2 | 86.58 | 15 | 0.31 | 0.54 | 0.04 | 0.12 | 59,81 |
| 66 | DL-Menthol | Decanoic acid | 1:1 | 164.27 | 11 | 2.43 | 3.11 | 0.37 | 0.69 | 82 |
| 67 | DL-Menthol | Octanoic acid | 1:1 | 150.24 | 11 | 2.72 | 3.43 | 0.41 | 0.90 | 82 |
| 68 | Dodecanoic acid | Decanoic acid | 1:2 | 181.61 | 10 | 3.10 | 3.67 | 1.78 | 2.42 | 82 |
| 69 | Dodecanoic acid | Octanoic acid | 1:3 | 158.24 | 11 | 3.49 | 4.13 | 1.69 | 2.41 | 82 |
| 70 | Ethylammonium bromide | Glycerol | 1:2 | 103.40 | 1 | 1.12 | 1.12 | 0.13 | 0.13 | 71 |
| 71 | Ethyl triphenylphosphonium iodide | Sulfolane | 1:4 | 179.79 | 5 | 0.35 | 0.40 | 0.06 | 0.15 | 83 |
| 72 | L-proline | DL-lactic acid | 1:1 | 102.61 | 11 | 1.00 | 1.43 | 0.19 | 0.35 | 43 |
| 73 | L-proline | Levulinic acid | 1:2 | 115.78 | 11 | 1.64 | 2.04 | 0.03 | 0.12 | 43 |
| 74 | Malonic acid | 1,4-butanediol | 1:1 | 97.09 | 1 | 1.88 | 1.88 | 3.60 | 3.60 | 78 |
| 75 | Methyl triphenylphosphunium bromide | Mono ethanolamine | 1:5 | 110.44 | 7 | 3.42 | 3.87 | 2.99 | 3.16 | 80 |
| 76 | Methyl triphenylphosphunium bromide | Mono ethanolamine | 1:6 | 103.39 | 7 | 0.37 | 0.77 | 0.31 | 0.49 | 80 |
| 77 | Methyl triphenylphosphunium bromide | Mono ethanolamine | 1:7 | 98.10 | 7 | 0.34 | 0.58 | 0.64 | 0.91 | 80 |
| 78 | Methyl triphenylphosphunium bromide | Mono ethanolamine | 1:8 | 93.99 | 7 | 0.30 | 0.51 | 0.79 | 0.99 | 80 |
| 79 | Methyl triphenylphosphunium bromide | Ethylene glycol | 1:3 | 135.86 | 1 | 3.31 | 3.31 | 1.17 | 1.17 | 73 |
| 80 | Methyl triphenylphosphunium bromide | Ethylene glycol | 1:4 | 121.10 | 1 | 3.59 | 3.59 | 1.31 | 1.31 | 73 |
| 81 | Methyl triphenylphosphunium bromide | Ethylene glycol | 1:5.25 | 109.29 | 1 | 3.31 | 3.31 | 1.34 | 1.34 | 73 |
| 82 | Methyl triphenylphosphunium bromide | Glycerol | 1:2 | 180.47 | 1 | 0.25 | 0.25 | 0.62 | 0.62 | 73 |
| 83 | Methyl triphenylphosphunium bromide | Glycerol | 1:3 | 158.38 | 1 | 4.45 | 4.45 | 0.10 | 0.10 | 73 |
| 84 | Methyl triphenylphosphunium bromide | Glycerol | 1:4 | 145.12 | 1 | 4.33 | 4.33 | 0.34 | 0.34 | 73 |
| 85 | Methyl triphenylphosphunium bromide | Triethylene glycol | 1:3 | 201.93 | 1 | 3.00 | 3.00 | 0.88 | 0.88 | 73 |
| 86 | Methyl triphenylphosphunium bromide | Triethylene glycol | 1:4 | 191.58 | 1 | 2.29 | 2.29 | 0.71 | 0.71 | 73 |
| 87 | Methyl triphenylphosphunium bromide | Triethylene glycol | 1:5 | 184.68 | 1 | 1.87 | 1.87 | 0.71 | 0.71 | 73 |
| 88 | N,N-diethyl ethanolammonium chloride | Ethylene glycol | 1:2 | 92.60 | 10 | 1.06 | 1.48 | 0.43 | 0.84 | 84 |
| 89 | N,N-diethyl ethanolammonium chloride | Ethylene glycol | 1:2.5 | 88.23 | 1 | 0.74 | 0.74 | 0.03 | 0.03 | 73 |
| 90 | N,N-diethyl ethanolammonium chloride | Ethylene glycol | 1:3 | 84.96 | 1 | 0.88 | 0.88 | 0.10 | 0.10 | 73 |
| 91 | N,N-diethyl ethanolammonium chloride | Ethylene glycol | 1:4 | 80.38 | 1 | 1.07 | 1.07 | 0.15 | 0.15 | 73 |
| 92 | N,N-diethyl ethanolammonium chloride | Glycerol | 1:2 | 112.61 | 11 | 0.45 | 0.79 | 0.38 | 0.68 | 73,84 |
| 93 | N,N-diethyl ethanolammonium chloride | Glycerol | 1:3 | 107.48 | 1 | 0.74 | 0.74 | 0.54 | 0.54 | 73 |
| 94 | N,N-diethyl ethanolammonium chloride | Glycerol | 1:4 | 104.41 | 1 | 0.76 | 0.76 | 0.48 | 0.48 | 73 |
| 95 | Propylammonium bromide | Glycerol | 1:2 | 108.07 | 1 | 1.08 | 1.08 | 0.16 | 0.16 | 71 |
| 96 | Tetra butylammonium chloride | Arginine | 6:1 | 263.10 | 6 | 0.28 | 0.34 | 0.19 | 0.25 | 85 |
| 97 | Tetra butylammonium chloride | Arginine | 7:1 | 264.95 | 6 | 0.20 | 0.24 | 0.10 | 0.25 | 85 |
| 98 | Tetra butylammonium chloride | Arginine | 8:1 | 266.39 | 6 | 0.07 | 0.11 | 0.35 | 0.60 | 85 |
| 99 | Tetra butylammonium chloride | Aspartic acid | 9:1 | 263.44 | 6 | 0.11 | 0.29 | 0.13 | 0.16 | 85 |
| 100 | Tetra butylammonium chloride | Aspartic acid | 10:1 | 264.75 | 6 | 0.15 | 0.31 | 0.17 | 0.31 | 85 |
| 101 | Tetra butylammonium chloride | Aspartic acid | 11:1 | 265.85 | 6 | 0.31 | 0.41 | 0.56 | 0.79 | 85 |
| 102 | Tetra butylammonium chloride | Ethylene glycol | 1:2 | 134.02 | 18 | 0.51 | 1.23 | 0.27 | 0.70 | 63,64 |
| 103 | Tetra butylammonium chloride | Ethylene glycol | 1:3 | 116.03 | 7 | 0.87 | 1.48 | 0.37 | 0.77 | 63 |
| 104 | Tetra butylammonium chloride | Ethylene glycol | 1:4 | 105.24 | 7 | 1.08 | 1.73 | 0.40 | 0.88 | 63 |
| 105 | Tetra butylammonium chloride | Glutamic acid | 8:1 | 263.39 | 6 | 0.12 | 0.27 | 0.37 | 0.41 | 85 |
| 106 | Tetra butylammonium chloride | Glutamic acid | 9:1 | 264.84 | 6 | 0.14 | 0.31 | 0.08 | 0.11 | 85 |
| 107 | Tetra butylammonium chloride | Glutamic acid | 10:1 | 266.03 | 6 | 0.17 | 0.30 | 0.17 | 0.31 | 85 |
| 108 | Tetra butylammonium chloride | Glycerol | 1:3 | 138.55 | 7 | 0.41 | 0.86 | 0.31 | 0.63 | 63 |
| 109 | Tetra butylammonium chloride | Glycerol | 1:4 | 129.26 | 7 | 0.35 | 0.73 | 0.24 | 0.40 | 63 |
| 110 | Tetra butylammonium chloride | Glycerol | 1:5 | 123.06 | 7 | 0.39 | 0.81 | 0.22 | 0.37 | 63 |
| 111 | Tetra butylammonium chloride | Levulinic acid | 1:1 | 197.01 | 11 | 1.59 | 2.09 | 0.43 | 0.79 | 39 |
| 112 | Tetra butylammonium bromide | Levulinic acid | 1:1 | 219.24 | 11 | 0.79 | 1.26 | 0.14 | 0.32 | 39 |
| 113 | Tetra butylammonium bromide | Monoethanolamine | 1:3 | 126.41 | 7 | 0.56 | 1.12 | 0.75 | 1.11 | 80 |
| 114 | Tetra butylammonium bromide | Monoethanolamine | 1:4 | 113.34 | 7 | 0.39 | 0.81 | 0.49 | 0.82 | 80 |
| 115 | Tetra butylammonium bromide | Monoethanolamine | 1:5 | 104.63 | 7 | 0.39 | 0.77 | 0.41 | 0.74 | 80 |
| 116 | Tetra butylammonium bromide | Monoethanolamine | 1:6 | 98.41 | 7 | 0.36 | 0.73 | 0.22 | 0.39 | 80 |
| 117 | Tetra butylammonium chloride | Phenylacetic acid | 1:2 | 146.00 | 11 | 0.89 | 1.18 | 0.09 | 0.17 | 64 |
| 118 | Tetra butylammonium chloride | Propionic acid | 1:2 | 142.03 | 11 | 1.48 | 1.96 | 0.29 | 0.68 | 64 |
| 119 | Tetra butylammonium chloride | Triethylene glycol | 1:1 | 214.04 | 7 | 0.40 | 0.82 | 0.49 | 0.99 | 39 |
| 120 | Tetra butylammonium chloride | Triethylene glycol | 2:1 | 235.33 | 7 | 0.83 | 1.33 | 1.55 | 1.98 | 39 |
| 121 | Tetra butylammonium chloride | Triethylene glycol | 3:1 | 245.98 | 7 | 0.97 | 1.39 | 1.81 | 2.11 | 39 |
| 122 | Tetra butylammonium chloride | Triethylene glycol | 4:1 | 252.37 | 7 | 1.01 | 1.43 | 1.81 | 2.08 | 39 |
| 123 | Tetra butylammonium chloride | L-methionine | 9:1 | 265.05 | 6 | 0.14 | 0.35 | 0.14 | 0.17 | 86 |
| 124 | Tetra butylammonium chloride | L-methionine | 10:1 | 266.22 | 6 | 0.09 | 0.24 | 0.05 | 0.16 | 86 |
| 125 | Tetra butylammonium chloride | L-methionine | 11:1 | 267.19 | 6 | 0.49 | 0.64 | 0.68 | 0.85 | 86 |
| 126 | Tetra butylammonium chloride | L-serine | 8:1 | 258.71 | 6 | 0.17 | 0.31 | 0.23 | 0.27 | 86 |
| 127 | Tetra butylammonium chloride | L-serine | 9:1 | 260.63 | 6 | 0.12 | 0.25 | 0.07 | 0.15 | 86 |
| 128 | Tetra butylammonium chloride | L-serine | 10:1 | 262.21 | 6 | 0.12 | 0.32 | 0.39 | 0.53 | 86 |
| 129 | Tetra butylammonium chloride | L-threonine | 8:1 | 260.27 | 6 | 0.27 | 0.51 | 0.28 | 0.31 | 86 |
| 130 | Tetra butylammonium chloride | L-threonine | 9:1 | 262.04 | 6 | 0.29 | 0.46 | 0.09 | 0.13 | 86 |
| 131 | Tetra butylammonium chloride | L-threonine | 10:1 | 263.48 | 6 | 0.12 | 0.24 | 0.56 | 0.81 | 86 |
| 132 | Tetra ethylammonium bromide | Levulinic acid | 1:1 | 163.14 | 11 | 1.22 | 1.70 | 0.14 | 0.27 | 11 |
| 133 | Tetra ethylammonium chloride | Levulinic acid | 1:1 | 140.91 | 11 | 1.56 | 2.05 | 0.18 | 0.32 | 11 |
| 134 | Tetra propylammonium bromide | Ethylene glycol | 1:3 | 113.12 | 7 | 0.47 | 1.04 | 0.36 | 0.81 | 87 |
| 135 | Tetra propylammonium bromide | Ethylene glycol | 1:4 | 102.91 | 7 | 0.69 | 1.31 | 0.46 | 0.94 | 87 |
| 136 | Tetra propylammonium bromide | Ethylene glycol | 1:5 | 96.10 | 7 | 0.93 | 1.58 | 0.59 | 1.10 | 87 |
| 137 | Tetra propylammonium bromide | Glycerol | 1:2 | 150.15 | 7 | 0.87 | 1.37 | 0.26 | 0.54 | 87 |
| 138 | Tetra propylammonium bromide | Glycerol | 1:3 | 135.64 | 7 | 0.74 | 1.27 | 0.20 | 0.35 | 87 |
| 139 | Tetra propylammonium bromide | Glycerol | 1:4 | 126.93 | 7 | 0.63 | 1.12 | 0.18 | 0.43 | 87 |
| 140 | Tetra propylammonium bromide | Triethylene glycol | 1:2.5 | 183.34 | 7 | 0.33 | 0.69 | 0.32 | 0.71 | 87 |
| 141 | Tetra propylammonium bromide | Triethylene glycol | 1:3 | 179.19 | 7 | 0.31 | 0.53 | 0.26 | 0.45 | 87 |
| 142 | Tetra propylammonium bromide | Triethylene glycol | 1:4 | 173.39 | 7 | 0.38 | 0.85 | 0.29 | 0.61 | 87 |

Table S3. The list of investigated DESs with the corresponding information for developing the heat capacity models, and the resulting values of *AARD%* and *maximum ARD%* from the AC and GC models for each individual DES.

| # | HBA | HBD | Molar Ratio HBA:HBD | Mw (g/mol) | No. of data points | AC results | | GC results | | Ref. |
| --- | --- | --- | --- | --- | --- | --- | --- | --- | --- | --- |
|  |  |  |  |  |  | AARD% | Max ARD% | AARD% | Max ARD% |  |
| 1 | Betaine | Ethylene glycol | 1:3 | 75.84 | 10 | 0.23 | 0.60 | 0.42 | 0.88 | 88 |
| 2 | Betaine | Glycerol | 1:3 | 98.36 | 1 | 40.09 | 40.09 | 47.72 | 47.72 | 70 |
| 3 | Betaine | Propylene glycol | 1:3.5 | 85.21 | 1 | 32.64 | 32.64 | 87.82 | 87.82 | 70 |
| 4 | Choline chloride | Citric acid | 1:2 | 174.62 | 23 | 22.30 | 23.99 | 0.26 | 0.70 | 89 |
| 5 | Choline chloride | Ethylene glycol | 1:2 | 87.92 | 14 | 8.78 | 9.06 | 2.50 | 3.20 | 90-92 |
| 6 | Choline chloride | D-fructose | 2:1 | 153.13 | 23 | 4.25 | 6.30 | 0.89 | 1.70 | 89 |
| 7 | Choline chloride | D-glucose | 2:1 | 153.13 | 23 | 0.55 | 1.29 | 0.47 | 1.11 | 89 |
| 8 | Choline chloride | Glycerol | 1:2 | 107.94 | 39 | 4.28 | 6.93 | 2.74 | 5.76 | 75,90-92 |
| 9 | Choline chloride | Malonic acid | 1:1 | 121.84 | 23 | 12.15 | 13.40 | 4.27 | 4.63 | 89 |
| 10 | Choline chloride | Oxalic acid | 1:2 | 106.56 | 23 | 9.94 | 11.99 | 32.97 | 35.45 | 89 |
| 11 | Choline chloride | Phenol | 1:3 | 105.49 | 23 | 0.53 | 1.65 | 0.50 | 1.17 | 89 |
| 12 | Choline chloride | Triethylene glycol | 1:2 | 146.65 | 23 | 7.13 | 9.39 | 0.57 | 0.92 | 89 |
| 13 | Choline chloride | Urea | 1:2 | 86.58 | 23 | 0.78 | 1.57 | 0.63 | 1.93 | 59,90,91 |
| 14 | L-cartinine | Ethylene glycol | 1:3 | 86.85 | 10 | 0.51 | 1.77 | 1.05 | 3.19 | 88 |
| 15 | Methyl triphenylphosphonium bromide | Ethylene glycol | 1:4 | 121.1 | 23 | 0.11 | 0.86 | 0.50 | 1.31 | 89 |
| 16 | Methyl triphenylphosphonium bromide | Glycerol | 1:3 | 158.38 | 23 | 23.40 | 24.17 | 1.51 | 2.83 | 89 |
| 17 | Methyl triphenylphosphonium bromide | Malonic acid | 2:3 | 205.32 | 23 | 1.03 | 2.87 | 1.31 | 3.27 | 89 |
| 18 | N,N-diethylethanolammonium chloride | Ethylene glycol | 1:2 | 92.6 | 11 | 3.42 | 3.63 | 0.57 | 1.62 | 93 |
| 19 | N,N-diethylethanolammonium chloride | Glycerol | 1:2 | 112.61 | 11 | 4.71 | 5.37 | 0.32 | 0.59 | 93 |
| 20 | Tetra butylammonium chloride | Ethylene glycol | 1:3 | 116.03 | 23 | 9.93 | 10.45 | 2.08 | 4.25 | 89 |
| 21 | Tetra butylammonium chloride | Glycerol | 1:5 | 123.07 | 23 | 35.90 | 36.50 | 0.58 | 1.55 | 89 |
| 22 | Tetra butylammonium chloride | Malonic acid | 1:3 | 147.52 | 23 | 1.92 | 5.91 | 2.54 | 7.42 | 89 |
| 23 | Tetra butylammonium chloride | Triethylene glycol | 1:1 | 214.04 | 23 | 46.95 | 47.44 | 2.88 | 5.86 | 89 |
| 24 | Tetra butylammonium chloride | Urea | 4:1 | 234.34 | 19 | 2.43 | 4.70 | 0.35 | 1.70 | 89 |

Table S4. The list of investigated DESs with the corresponding information for developing the speed of sound models, and the resulting values of *AARD%* and *maximum ARD%* from the AC and GC models for each individual DES.

| # | HBA | HBD | Molar Ratio HBA:HBD | Mw (g/mol) | No. of data points | AC results | | GC results | | Ref. |
| --- | --- | --- | --- | --- | --- | --- | --- | --- | --- | --- |
|  |  |  |  |  |  | AARD% | Max ARD% | AARD% | Max ARD% |  |
| 1 | 1-Ethyl-3-methylimidazolium chloride | Ethylene glycol | 2:1 | 118.44 | 5 | 12.90 | 13.02 | 0.06 | 0.10 | [94](file:///C:\Users\Reza\Desktop\GCDES%20working%20files\AARD%25%20and%20MaxAARD%25.xlsx#RANGE!_ENREF_30) |
| 2 | 1-Ethyl-3-methylimidazolium chloride | Ethylene glycol | 1:1 | 104.34 | 5 | 1.01 | 1.93 | 0.67 | 1.45 | [94](file:///C:\Users\Reza\Desktop\GCDES%20working%20files\AARD%25%20and%20MaxAARD%25.xlsx#RANGE!_ENREF_30) |
| 3 | 1-Ethyl-3-methylimidazolium chloride | Ethylene glycol | 1:2 | 90.25 | 5 | 4.07 | 4.63 | 3.56 | 4.27 | [94](file:///C:\Users\Reza\Desktop\GCDES%20working%20files\AARD%25%20and%20MaxAARD%25.xlsx#RANGE!_ENREF_30) |
| 4 | Benzyl tributylammonium chloride | Diethylene glycol | 1:3 | 157.57 | 13 | 1.35 | 2.89 | 0.51 | 0.90 | [67](file:///C:\Users\Reza\Desktop\GCDES%20working%20files\AARD%25%20and%20MaxAARD%25.xlsx#RANGE!_ENREF_31) |
| 5 | Benzyl tributylammonium chloride | Ethylene glycol | 1:3 | 124.53 | 13 | 1.59 | 1.69 | 0.92 | 0.98 | [67](file:///C:\Users\Reza\Desktop\GCDES%20working%20files\AARD%25%20and%20MaxAARD%25.xlsx#RANGE!_ENREF_31) |
| 6 | Benzyl tributylammonium chloride | Glycerol | 1:3 | 147.05 | 13 | 4.81 | 6.31 | 1.78 | 2.48 | [67](file:///C:\Users\Reza\Desktop\GCDES%20working%20files\AARD%25%20and%20MaxAARD%25.xlsx#RANGE!_ENREF_31) |
| 7 | Benzyl tributylammonium chloride | Triethylene glycol | 1:3 | 190.61 | 13 | 1.99 | 4.40 | 1.06 | 1.53 | [67](file:///C:\Users\Reza\Desktop\GCDES%20working%20files\AARD%25%20and%20MaxAARD%25.xlsx#RANGE!_ENREF_31) |
| 8 | Benzyl trimethylammonium chloride | Diethylene glycol | 1:3 | 126.01 | 13 | 2.46 | 3.80 | 0.62 | 1.24 | [67](file:///C:\Users\Reza\Desktop\GCDES%20working%20files\AARD%25%20and%20MaxAARD%25.xlsx#RANGE!_ENREF_31) |
| 9 | Benzyl trimethylammonium chloride | Ethylene glycol | 1:3 | 92.97 | 13 | 0.17 | 0.26 | 2.10 | 2.66 | [67](file:///C:\Users\Reza\Desktop\GCDES%20working%20files\AARD%25%20and%20MaxAARD%25.xlsx#RANGE!_ENREF_31) |
| 10 | Benzyl trimethylammonium chloride | Glycerol | 1:3 | 115.49 | 13 | 6.02 | 6.98 | 1.19 | 2.85 | [67](file:///C:\Users\Reza\Desktop\GCDES%20working%20files\AARD%25%20and%20MaxAARD%25.xlsx#RANGE!_ENREF_31) |
| 11 | Benzyl trimethylammonium chloride | Triethylene glycol | 1:3 | 159.05 | 13 | 1.83 | 4.07 | 0.09 | 0.21 | [67](file:///C:\Users\Reza\Desktop\GCDES%20working%20files\AARD%25%20and%20MaxAARD%25.xlsx#RANGE!_ENREF_31) |
| 12 | Benzyl tripropylammonium chloride | Ethylene glycol | 1:3 | 114.02 | 11 | 0.11 | 0.22 | 1.18 | 1.24 | [42](file:///C:\Users\Reza\Desktop\GCDES%20working%20files\AARD%25%20and%20MaxAARD%25.xlsx#RANGE!_ENREF_32) |
| 13 | Benzyl tripropylammonium chloride | Glycerol | 1:3 | 136.53 | 11 | 5.83 | 6.49 | 0.38 | 0.74 | [42](file:///C:\Users\Reza\Desktop\GCDES%20working%20files\AARD%25%20and%20MaxAARD%25.xlsx#RANGE!_ENREF_32) |
| 14 | Benzyl tripropylammonium chloride | Lactic acid | 1:3 | 135.02 | 11 | 9.03 | 9.29 | 0.68 | 1.91 | [42](file:///C:\Users\Reza\Desktop\GCDES%20working%20files\AARD%25%20and%20MaxAARD%25.xlsx#RANGE!_ENREF_32) |
| 15 | Benzyl tripropylammonium chloride | Phenol | 1:3 | 138.05 | 11 | 0.16 | 0.57 | 0.14 | 0.51 | [42](file:///C:\Users\Reza\Desktop\GCDES%20working%20files\AARD%25%20and%20MaxAARD%25.xlsx#RANGE!_ENREF_32) |
| 16 | Betaine | Lactic acid | 1:2 | 99.10 | 10 | 6.30 | 6.91 | 0.45 | 1.19 | [43](file:///C:\Users\Reza\Desktop\GCDES%20working%20files\AARD%25%20and%20MaxAARD%25.xlsx#RANGE!_ENREF_21) |
| 17 | Betaine | Lactic acid | 1:5 | 94.59 | 11 | 2.38 | 4.79 | 1.50 | 3.35 | [43](file:///C:\Users\Reza\Desktop\GCDES%20working%20files\AARD%25%20and%20MaxAARD%25.xlsx#RANGE!_ENREF_21) |
| 18 | Betaine | Levulinic acid | 1:2 | 116.46 | 11 | 3.70 | 4.67 | 1.10 | 2.82 | [43](file:///C:\Users\Reza\Desktop\GCDES%20working%20files\AARD%25%20and%20MaxAARD%25.xlsx#RANGE!_ENREF_21) |
| 19 | Choline chloride | 1,2-propanediol | 1:3 | 91.98 | 10 | 5.20 | 5.52 | 1.10 | 2.47 | [72](file:///C:\Users\Reza\Desktop\GCDES%20working%20files\AARD%25%20and%20MaxAARD%25.xlsx#RANGE!_ENREF_37) |
| 20 | Choline chloride | Ethylene glycol | 1:2 | 87.92 | 13 | 2.30 | 5.82 | 1.36 | 3.63 | 95,96 |
| 21 | Choline chloride | Fructose | 2:1 | 153.13 | 7 | 9.57 | 11.30 | 0.47 | 1.07 | [96](file:///C:\Users\Reza\Desktop\GCDES%20working%20files\AARD%25%20and%20MaxAARD%25.xlsx#RANGE!_ENREF_34) |
| 22 | Choline chloride | Glucose | 2:1 | 153.13 | 7 | 15.28 | 27.93 | 10.98 | 20.04 | [96](file:///C:\Users\Reza\Desktop\GCDES%20working%20files\AARD%25%20and%20MaxAARD%25.xlsx#RANGE!_ENREF_34) |
| 23 | Choline chloride | Glutaric acid | 1:1 | 135.87 | 7 | 0.51 | 1.18 | 0.98 | 1.86 | [97](file:///C:\Users\Reza\Desktop\GCDES%20working%20files\AARD%25%20and%20MaxAARD%25.xlsx#RANGE!_ENREF_39) |
| 24 | Choline chloride | Glycerol | 1:2 | 107.94 | 38 | 7.31 | 15.05 | 3.40 | 18.25 | 75,95,96 |
| 25 | Choline chloride | Levulinic acid | 1:2 | 123.95 | 11 | 6.49 | 7.17 | 0.50 | 1.08 | [77](file:///C:\Users\Reza\Desktop\GCDES%20working%20files\AARD%25%20and%20MaxAARD%25.xlsx#RANGE!_ENREF_38) |
| 26 | Choline chloride | Malonic acid | 1:1 | 121.84 | 7 | 0.75 | 1.55 | 0.63 | 1.25 | [97](file:///C:\Users\Reza\Desktop\GCDES%20working%20files\AARD%25%20and%20MaxAARD%25.xlsx#RANGE!_ENREF_39) |
| 27 | Choline chloride | Oxalic acid | 1:1 | 114.83 | 4 | 0.89 | 1.23 | 0.27 | 0.44 | [98](file:///C:\Users\Reza\Desktop\GCDES%20working%20files\AARD%25%20and%20MaxAARD%25.xlsx#RANGE!_ENREF_40) |
| 28 | Choline chloride | Urea | 1:2 | 86.58 | 20 | 1.49 | 4.96 | 1.92 | 5.54 | 95,96,99 |
| 29 | Dodecanoic acid | Decanoic acid | 1:2 | 181.61 | 10 | 0.24 | 0.48 | 3.67 | 6.64 | [82](file:///C:\Users\Reza\Desktop\GCDES%20working%20files\AARD%25%20and%20MaxAARD%25.xlsx#RANGE!_ENREF_41) |
| 30 | Dodecanoic acid | Octanoic acid | 1:3 | 158.24 | 11 | 0.45 | 1.14 | 2.26 | 4.24 | [82](file:///C:\Users\Reza\Desktop\GCDES%20working%20files\AARD%25%20and%20MaxAARD%25.xlsx#RANGE!_ENREF_41) |
| 31 | Menthol | Camphor-10-sulfonic acid | 5:1 | 168.94 | 5 | 22.64 | 24.74 | 1.72 | 2.87 | [100](file:///C:\Users\Reza\Desktop\GCDES%20working%20files\AARD%25%20and%20MaxAARD%25.xlsx#RANGE!_ENREF_42) |
| 32 | Menthol | Decanoic acid | 1:1 | 164.27 | 11 | 8.14 | 11.41 | 1.93 | 4.06 | [82](file:///C:\Users\Reza\Desktop\GCDES%20working%20files\AARD%25%20and%20MaxAARD%25.xlsx#RANGE!_ENREF_41) |
| 33 | Menthol | Ethylene glycol | 1:1 | 109.17 | 5 | 5.47 | 6.79 | 1.72 | 2.54 | [100](file:///C:\Users\Reza\Desktop\GCDES%20working%20files\AARD%25%20and%20MaxAARD%25.xlsx#RANGE!_ENREF_42) |
| 34 | Menthol | Octanoic acid | 1:1 | 150.24 | 11 | 11.59 | 15.60 | 1.60 | 3.80 | [82](file:///C:\Users\Reza\Desktop\GCDES%20working%20files\AARD%25%20and%20MaxAARD%25.xlsx#RANGE!_ENREF_41) |
| 35 | Menthol | Salicylic acid | 4:1 | 152.64 | 5 | 11.71 | 12.86 | 1.08 | 1.88 | [100](file:///C:\Users\Reza\Desktop\GCDES%20working%20files\AARD%25%20and%20MaxAARD%25.xlsx#RANGE!_ENREF_42) |
| 36 | Proline | Lactic acid | 1:1 | 102.61 | 10 | 5.66 | 9.85 | 2.03 | 5.92 | [43](file:///C:\Users\Reza\Desktop\GCDES%20working%20files\AARD%25%20and%20MaxAARD%25.xlsx#RANGE!_ENREF_21) |
| 37 | Proline | Levulinic acid | 1:2 | 115.78 | 11 | 0.91 | 1.70 | 1.57 | 3.46 | [43](file:///C:\Users\Reza\Desktop\GCDES%20working%20files\AARD%25%20and%20MaxAARD%25.xlsx#RANGE!_ENREF_21) |

Table S5. The list of investigated DESs with the corresponding information for developing the surface tension models, and the resulting values of *AARD%* and *maximum ARD%* from the AC and GC models for each individual DES.

| # | HBA | HBD | Molar Ratio HBA:HBD | Mw (g/mol) | No. of data points | AC results | | GC results | | Ref. |
| --- | --- | --- | --- | --- | --- | --- | --- | --- | --- | --- |
|  |  |  |  |  |  | AARD% | Max ARD% | AARD% | Max ARD% |  |
| 1 | Acetylcholine chloride | Glycerol | 1:2 | 121.95 | 1 | 20.70 | 20.70 | 24.13 | 24.13 | 101 |
| 2 | Acetylcholine chloride | Urea | 1:2 | 100.59 | 1 | 14.34 | 14.34 | 8.93 | 8.93 | 101 |
| 3 | Allyl triphenylphosphonium bromide | Diethylene glycol | 1:4 | 161.54 | 10 | 2.37 | 5.38 | 2.19 | 4.55 | 102 |
| 4 | Allyl triphenylphosphonium bromide | Diethylene glycol | 1:10 | 131.31 | 10 | 2.68 | 5.10 | 0.51 | 1.04 | 102 |
| 5 | Allyl triphenylphosphonium bromide | Diethylene glycol | 1:16 | 122.42 | 10 | 2.23 | 4.85 | 0.66 | 0.96 | 102 |
| 6 | Allyl triphenylphosphonium bromide | Glycerol | 1:14 | 111.51 | 4 | 51.92 | 57.97 | 42.76 | 47.49 | 103 |
| 7 | Allyl triphenylphosphonium bromide | Triethylene glycol | 1:4 | 196.79 | 10 | 3.29 | 7.75 | 2.15 | 4.49 | 102 |
| 8 | Allyl triphenylphosphonium bromide | Triethylene glycol | 1:10 | 171.36 | 10 | 2.03 | 4.32 | 0.68 | 1.35 | 102 |
| 9 | Allyl triphenylphosphonium bromide | Triethylene glycol | 1:16 | 163.88 | 10 | 9.55 | 9.69 | 2.52 | 5.35 | 102 |
| 10 | Benzyl triphenylphosphonium chloride | Ethylene glycol | 1:3 | 89.3 | 1 | 3.20 | 3.20 | 19.00 | 19.00 | 104 |
| 11 | Benzyl triphenylphosphonium chloride | Ethylene glycol | 1:11 | 89.3 | 5 | 14.83 | 27.33 | 24.14 | 38.68 | 104 |
| 12 | Benzyl triphenylphosphonium chloride | Glycerol | 1:16 | 109.55 | 7 | 18.18 | 22.16 | 1.02 | 2.43 | 103,105 |
| 13 | Butylammonium bromide | Glycerol | 1:2 | 112.75 | 1 | 4.72 | 4.72 | 8.57 | 8.57 | 71 |
| 14 | Choline chloride | 1,4-butanediol | 1:3 | 102.5 | 1 | 0.21 | 0.21 | 18.95 | 18.95 | 106 |
| 15 | Choline chloride | 1,5-pentandiol | 1:3.5 | 112.03 | 1 | 9.84 | 9.84 | 43.52 | 43.52 | 107 |
| 16 | Choline chloride | Citric acid monohydrate | 1:1 | 174.88 | 1 | 49.80 | 49.80 | 15.22 | 15.22 | 108 |
| 17 | Choline chloride | Citric acid monohydrate | 1:2 | 186.63 | 1 | 69.39 | 69.39 | 0.33 | 0.33 | 108 |
| 18 | Choline chloride | Citric acid monohydrate | 1:3 | 192.51 | 1 | 89.14 | 89.14 | 15.45 | 15.45 | 108 |
| 19 | Choline chloride | Citric acid monohydrate | 2:1 | 163.13 | 1 | 80.97 | 80.97 | 51.94 | 51.94 | 108 |
| 20 | Choline chloride | Citric acid monohydrate | 3:1 | 157.25 | 1 | 129.19 |  | 103.89 | 103.89 | 108 |
| 21 | Choline chloride | D-fructose | 1:1 | 159.89 | 7 | 11.98 | 19.15 | 13.60 | 20.39 | 47 |
| 23 | Choline chloride | D-fructose | 1.5:1 | 155.83 | 7 | 7.10 | 14.18 | 6.91 | 13.79 | 47 |
| 22 | Choline chloride | D-fructose | 2:1 | 153.13 | 7 | 4.93 | 10.41 | 5.30 | 12.17 | 47 |
| 24 | Choline chloride | D-fructose | 2.5:1 | 151.2 | 7 | 9.15 | 18.33 | 12.66 | 21.88 | 47 |
| 25 | Choline chloride | D-glucose | 1:1 | 159.89 | 7 | 21.15 | 22.02 | 15.30 | 16.03 | 109 |
| 27 | Choline chloride | D-glucose | 1.5:1 | 155.83 | 7 | 11.94 | 12.92 | 4.43 | 5.29 | 109 |
| 26 | Choline chloride | D-glucose | 2:1 | 153.13 | 7 | 1.56 | 3.22 | 7.73 | 9.00 | 110 |
| 28 | Choline chloride | D-glucose | 2.5:1 | 151.2 | 7 | 2.80 | 4.66 | 13.29 | 15.16 | 109 |
| 29 | Choline chloride | Ethylene glycol | 1:2 | 87.92 | 7 | 11.60 | 14.66 | 12.05 | 15.02 | 110 |
| 30 | Choline chloride | Glycerol | 1:2 | 107.94 | 32 | 14.78 | 19.50 | 14.34 | 18.94 | 101,110 |
| 31 | Choline chloride | Lactic acid | 1:1 | 114.85 | 1 | 28.07 | 28.07 | 62.46 | 62.46 | 111 |
| 32 | Choline chloride | Levulinic acid | 1:2 | 123.95 | 1 | 40.60 | 40.60 | 0.37 | 0.37 | 112 |
| 33 | Choline chloride | Malonic acid | 1:1 | 121.84 | 1 | 12.01 | 12.01 | 15.69 | 15.69 | 113 |
| 34 | Choline chloride | Monoethanolamine | 1:5 | 74.17 | 7 | 5.34 | 8.55 | 7.43 | 10.41 | 80 |
| 35 | Choline chloride | Monoethanolamine | 1:6 | 72.3 | 7 | 2.78 | 5.85 | 4.98 | 7.88 | 80 |
| 36 | Choline chloride | Monoethanolamine | 1:7 | 70.9 | 7 | 1.72 | 3.43 | 2.58 | 5.62 | 80 |
| 37 | Choline chloride | Monoethanolamine | 1:8 | 69.81 | 7 | 2.27 | 5.06 | 1.69 | 3.62 | 80 |
| 38 | Choline chloride | Oxalic acid dihydrate | 1:1 | 132.85 | 1 | 1.78 | 1.78 | 4.75 | 4.75 | 107 |
| 39 | Choline chloride | Phenol | 1:2 | 109.28 | 1 | 45.13 | 45.13 | 1.24 | 1.24 | 112 |
| 40 | Choline chloride | Phenylacetic acid | 1:2 | 137.31 | 1 | 27.14 | 27.14 | 45.12 | 45.12 | 113 |
| 41 | Choline chloride | Urea | 1:2 | 86.58 | 3 | 6.52 | 28.15 | 40.91 | 51.71 | 101,114,115 |
| 42 | Ethylammonium bromide | Glycerol | 1:2 | 103.4 | 1 | 1.35 | 1.35 | 19.92 | 19.92 | 71 |
| 43 | Methyl triphenylphosphonium bromide | Ethylene glycol | 1:3 | 135.86 | 6 | 17.14 | 28.15 | 16.35 | 30.65 | 104 |
| 44 | Methyl triphenylphosphonium bromide | Ethylene glycol | 1:4 | 121.1 | 7 | 8.32 | 11.09 | 11.81 | 14.30 | 110 |
| 45 | Methyl triphenylphosphonium bromide | Glycerol | 1:3 | 158.38 | 7 | 10.15 | 12.20 | 7.03 | 8.90 | 110 |
| 46 | Methyl triphenylphosphonium bromide | Monoethanolamine | 1:5 | 110.44 | 7 | 2.68 | 5.78 | 3.09 | 6.30 | 80 |
| 47 | Methyl triphenylphosphonium bromide | Monoethanolamine | 1:6 | 103.39 | 7 | 4.65 | 8.01 | 1.87 | 4.01 | 80 |
| 48 | Methyl triphenylphosphonium bromide | Monoethanolamine | 1:7 | 98.1 | 7 | 7.45 | 11.10 | 2.39 | 4.94 | 80 |
| 49 | Methyl triphenylphosphonium bromide | Monoethanolamine | 1:8 | 93.99 | 7 | 10.20 | 12.72 | 4.31 | 6.31 | 80 |
| 50 | Methyl triphenylphosphonium bromide | Triethylene glycol | 1:5 | 184.68 | 1 | 5.63 | 5.63 | 2.93 | 2.93 | 106 |
| 51 | N,N-diethylethanolammonium chloride | 2,2,2-Trifluoroacetamide | 1:2 | 126.58 | 1 | 0.01 | 0.01 | 2.37 | 2.37 | 106 |
| 52 | N,N-diethylethanolammonium chloride | Ethylene glycol | 1:3 | 84.96 | 7 | 4.90 | 7.87 | 3.40 | 6.16 | 110 |
| 53 | N,N-diethylethanolammonium chloride | Glycerol | 1:2 | 112.61 | 6 | 2.55 | 5.57 | 2.10 | 4.14 | 103 |
| 54 | N,N-diethylethanolammonium chloride | Glycerol | 1:4 | 104.41 | 7 | 3.82 | 6.51 | 5.17 | 7.64 | 110 |
| 55 | Propylammonium bromide | Glycerol | 1:2 | 108.07 | 1 | 7.96 | 7.96 | 15.69 | 15.69 | 71 |
| 56 | Tetra butylammonium bromide | Ethylene glycol | 1:3 | 148.83 | 7 | 14.58 | 14.89 | 23.41 | 24.21 | 104 |
| 57 | Tetra butylammonium bromide | Glycerol | 1:4 | 138.15 | 1 | 21.37 | 21.37 | 10.21 | 10.21 | 105 |
| 58 | Tetra butylammonium bromide | Monoethanolamine | 1:3 | 126.41 | 7 | 11.32 | 13.54 | 1.07 | 2.39 | 80 |
| 59 | Tetra butylammonium bromide | Monoethanolamine | 1:4 | 113.34 | 7 | 8.35 | 9.91 | 1.62 | 2.21 | 80 |
| 60 | Tetra butylammonium bromide | Monoethanolamine | 1:5 | 104.63 | 7 | 5.19 | 6.02 | 4.40 | 4.54 | 80 |
| 61 | Tetra butylammonium bromide | Monoethanolamine | 1:6 | 98.41 | 7 | 2.47 | 3.02 | 6.78 | 7.33 | 80 |
| 62 | Tetra butylammonium chloride | Arginine | 6:1 | 263.1 | 5 | 3.42 | 4.23 | 1.26 | 2.34 | 116 |
| 63 | Tetra butylammonium chloride | Arginine | 7:1 | 264.95 | 5 | 1.53 | 2.73 | 2.96 | 6.67 | 116 |
| 64 | Tetra butylammonium chloride | Arginine | 8:1 | 266.39 | 5 | 5.08 | 7.07 | 1.88 | 3.68 | 116 |
| 65 | Tetra butylammonium chloride | Aspartic acid | 9:1 | 263.44 | 5 | 5.84 | 6.61 | 1.42 | 3.68 | 116 |
| 66 | Tetra butylammonium chloride | Aspartic acid | 10:1 | 264.75 | 5 | 0.86 | 1.51 | 3.60 | 4.07 | 116 |
| 67 | Tetra butylammonium chloride | Aspartic acid | 11:1 | 265.85 | 5 | 5.24 | 7.48 | 7.87 | 8.35 | 116 |
| 68 | Tetra butylammonium chloride | Ethylene glycol | 1:2 | 134.02 | 6 | 7.99 | 13.09 | 3.62 | 7.55 | 63 |
| 69 | Tetra butylammonium chloride | Ethylene glycol | 1:3 | 116.03 | 7 | 4.51 | 9.40 | 2.46 | 4.06 | 63 |
| 70 | Tetra butylammonium chloride | Ethylene glycol | 1:4 | 105.24 | 6 | 3.55 | 7.32 | 2.30 | 4.87 | 63 |
| 71 | Tetra butylammonium chloride | Glutamic acid | 8:1 | 263.39 | 5 | 1.15 | 3.51 | 8.61 | 11.54 | 116 |
| 72 | Tetra butylammonium chloride | Glutamic acid | 9:1 | 264.84 | 5 | 15.54 | 19.13 | 5.70 | 11.20 | 116 |
| 73 | Tetra butylammonium chloride | Glutamic acid | 10:1 | 266.03 | 5 | 5.08 | 6.14 | 3.19 | 5.08 | 116 |
| 74 | Tetra butylammonium chloride | Glycerol | 1:3 | 138.55 | 6 | 4.37 | 7.67 | 8.63 | 11.37 | 63 |
| 75 | Tetra butylammonium chloride | Glycerol | 1:4 | 129.26 | 6 | 4.54 | 7.33 | 8.97 | 11.15 | 63 |
| 76 | Tetra butylammonium chloride | Glycerol | 1:5 | 123.06 | 6 | 4.27 | 6.52 | 8.88 | 10.48 | 63 |
| 77 | Tetra butylammonium chloride | L-methionine | 9:1 | 265.05 | 5 | 1.70 | 3.08 | 2.38 | 4.92 | 86 |
| 78 | Tetra butylammonium chloride | L-methionine | 10:1 | 266.22 | 5 | 6.03 | 7.79 | 4.73 | 7.85 | 86 |
| 79 | Tetra butylammonium chloride | L-methionine | 11:1 | 267.19 | 5 | 1.64 | 2.83 | 3.11 | 6.67 | 86 |
| 80 | Tetra butylammonium chloride | L-serine | 8:1 | 258.71 | 5 | 0.37 | 0.81 | 1.17 | 2.47 | 86 |
| 81 | Tetra butylammonium chloride | L-serine | 9:1 | 260.63 | 5 | 1.65 | 3.16 | 2.71 | 5.85 | 86 |
| 82 | Tetra butylammonium chloride | L-serine | 10:1 | 262.21 | 5 | 5.33 | 7.49 | 3.32 | 6.66 | 86 |
| 83 | Tetra butylammonium chloride | L-threonine | 8:1 | 260.27 | 6 | 1.76 | 3.72 | 44.21 | 45.11 | 86 |
| 84 | Tetra butylammonium chloride | L-threonine | 9:1 | 262.04 | 6 | 3.36 | 5.17 | 38.60 | 39.34 | 86 |
| 85 | Tetra butylammonium chloride | L-threonine | 10:1 | 263.48 | 6 | 3.87 | 5.52 | 50.79 | 51.71 | 86 |
| 86 | Tetra butylammonium chloride | Triethylene glycol | 1:1 | 214.04 | 6 | 5.95 | 11.56 | 3.68 | 7.59 | 63 |
| 87 | Tetra butylammonium chloride | Triethylene glycol | 2:1 | 235.33 | 6 | 3.93 | 7.69 | 2.19 | 4.51 | 63 |
| 88 | Tetra butylammonium chloride | Triethylene glycol | 3:1 | 245.98 | 7 | 1.50 | 2.87 | 1.63 | 3.51 | 63 |
| 89 | Tetra butylammonium chloride | Triethylene glycol | 4:1 | 252.37 | 6 | 3.90 | 4.72 | 4.50 | 5.39 | 63 |
| 90 | Tetra propylammonium bromide | Ethylene glycol | 1:3 | 113.12 | 6 | 2.89 | 6.52 | 6.73 | 10.76 | 87 |
| 91 | Tetra propylammonium bromide | Ethylene glycol | 1:4 | 102.91 | 7 | 2.67 | 5.78 | 5.05 | 9.60 | 87,105 |
| 92 | Tetra propylammonium bromide | Ethylene glycol | 1:5 | 96.1 | 6 | 2.38 | 5.10 | 4.85 | 8.50 | 87 |
| 93 | Tetra propylammonium bromide | Glycerol | 1:2 | 150.15 | 6 | 4.35 | 8.50 | 2.66 | 4.66 | 87 |
| 94 | Tetra propylammonium bromide | Glycerol | 1:3 | 135.64 | 7 | 7.35 | 10.40 | 3.48 | 6.39 | 105,87 |
| 95 | Tetra propylammonium bromide | Glycerol | 1:4 | 126.93 | 6 | 6.75 | 9.66 | 3.10 | 5.87 | 87 |
| 96 | Tetra propylammonium bromide | Triethylene glycol | 1:2.5 | 183.34 | 6 | 2.87 | 5.89 | 4.51 | 7.98 | 87 |
| 97 | Tetra propylammonium bromide | Triethylene glycol | 1:3 | 179.19 | 7 | 3.54 | 6.40 | 3.85 | 6.98 | 87,105 |
| 98 | Tetra propylammonium bromide | Triethylene glycol | 1:4 | 173.39 | 6 | 4.32 | 6.02 | 3.75 | 5.06 | 87 |

Table S6. Comparison of the values of *AARD%* for the GC and AC models for different groups of DESs with a common (fixed) HBA compound.

| **DESs with fixed HBA** | **AARD%** | | **Number of data points** |
| --- | --- | --- | --- |
|  | **GC** | **AC** |  |
| **Density** | | | |
| Choline chloride | 1.26 | 2.57 | 439 |
| Tetrabutylammonium chloride | 3.05 | 3.48 | 146 |
| Acetyl choline chloride | 0.60 | 1.85 | 120 |
| N,N diethylenethanol ammonium chloride | 1.12 | 2.96 | 110 |
| Methyl triphenylphosphonium bromide | 0.92 | 1.55 | 104 |
| Allyl triphenylphosphonium bromide | 1.04 | 1.84 | 66 |
| Benzyldimethyl(2-hydroxyethyl) ammonium chloride | 0.81 | 1.07 | 65 |
| Benzyl tripropyl ammonium chloride | 3.38 | 2.61 | 56 |
| **Refractive index** | | | |
| Choline chloride | 0.28 | 0.99 | 307 |
| Tetra butyl ammonium chloride | 0.41 | 0.53 | 222 |
| Benzyl trialkyl ammonium Chloride (n=1,2,3,4) | 0.30 | 0.99 | 202 |
| Allyltriphenyl phosphonium bromide | 0.14 | 1.20 | 66 |
| **Heat capacity** | | | |
| Choline chloride | 4.53 | 6.82 | 237 |
| Tetrabutylammonium chloride | 1.73 | 20.04 | 111 |
| Methyl triphenyl phosphonium bromide | 1.11 | 8.18 | 69 |
| **Speed of sound** | | | |
| Benzyl trialkyl ammonium chloride (n=1,3,4) | 0.90 | 2.90 | 148 |
| Choline chloride | 2.37 | 5.22 | 124 |
| **Surface tension** | | | |
| Tetra butylammonium chloride | 7.92 | 4.06 | 155 |
| Choline chloride | 10.55 | 12.49 | 139 |
| Tetra alkylammonium bromide (n=3,4) | 4.85 | 5.93 | 93 |
| Allyl triphenyl phosphonium bromide | 4.30 | 6.70 | 64 |

Table S7. Comparison of the values of *AARD%* for the GC and AC models for different groups of DESs with a common (fixed) HBD compound.

| **DESs with fixed HBD** | **AARD%** | | **Number of data points** |
| --- | --- | --- | --- |
|  | **GC** | **AC** |  |
| **Density** | | | |
| Ethylene glycol | 1.90 | 2.13 | 225 |
| Glycerol | 1.34 | 3.44 | 213 |
| Levulinic acid | 1.33 | 3.09 | 95 |
| Triethylene glycol | 1.68 | 2.47 | 68 |
| **Refractive index** | | | |
| Glycerol | 0.24 | 0.84 | 159 |
| Ethylene glycol | 0.39 | 0.93 | 122 |
| Triethylene glycol | 0.53 | 0.81 | 111 |
| Levulinic acid | 0.23 | 1.55 | 101 |
| Monoethanolamine | 0.64 | 0.70 | 84 |
| **Heat capacity** | | | |
| Glycerol | 2.13 | 16.73 | 97 |
| Ethylene glycol | 1.27 | 4.38 | 91 |
| Malonic acid | 2.72 | 5.03 | 68 |
| **Speed of sound** | | | |
| Glycerol | 2.29 | 6.43 | 75 |
| Ethylene glycol | 1.43 | 2.45 | 70 |
| **Surface tension** | | | |
| Glycerol | 12.7 | 11.12 | 104 |
| Monoethanolamine | 2.60 | 5.37 | 84 |
| Ethylene glycol | 7.83 | 7.78 | 78 |
| Triethylene glycol | 1.72 | 4.21 | 75 |

**Appendix A**

In this section we present example calculations by the proposed GC and AC models for density, refractive index, heat capacity, speed of sound and surface tension for the DESs choline chloride : glycerol (1:2 & 1:3) at a temperature of 298.15 K.

As the first step, the mole numbers of HBA and HBD should be normalized. For these DES, the smaller mole number is already equal to one (for the HBA, choline chloride), therefore, it is not necessary to normalize the mole numbers. Then for 1 ChCl: 2 Glycerol, m_HBA_=1 and m_HBD_=2 and for1 ChCl: 3 Glycerol, m_HBA_=1 and m_HBD_=3

In the next step the HBA and HBD molecules should be decomposed to their constituting functional groups for the GC models according to Table 7, and constituting atoms for the AC models according to Table 8, and based on the values of the resulting contributions, the summations should be calculated for each physical property. The decomposed functional groups and atoms, with the corresponding contribution summation results are presented in Tables A1 and A2 for the HBA and HBD of the GC models, respectively, and Tables A3 and A4 for the HBA and HBD of the AC models, respectively.

Table A6. List of the decomposed groups of choline chloride as the HBA of the GC models for the various properties.

| **Group** | *k_i_* | **Density** | | | **Refractive index** | | **Heat capacity** | | **Speed of sound** | | **Surface tension** | |
| --- | --- | --- | --- | --- | --- | --- | --- | --- | --- | --- | --- | --- |
|  |  | $\Delta\rho_{1}^{G}$ | $\Delta\rho_{2}^{G}$ | | $\Delta n_{1}^{G}$ | $\Delta n_{2}^{G}$ | $\Delta Cp_{1}^{G}$ | $\Delta Cp_{2}^{G}$ | $\Delta u_{1}^{G}$ | $\Delta u_{2}^{G}$ | $\Delta\sigma_{1}^{G}$ | $\Delta\sigma_{2}^{G}$ |
| **Without Ring** |  |  | |  |  |  |  |  |  |  |  |  |
| $-CH_{3}$ | 3 | 61.91 | 1.53 | | 160.09 | 5.70 | 85.44 | -445.78 | 381.49 | 164.90 | 19.99 | 8.31 |
| $-CH_{2}-$ | 2 | 41.87 | 8.27 | | 19.02 | 5.80 | 1.13 | 159.25 | 36.98 | 16.26 | 16.77 | 17.40 |
| $OH$ | 1 | -30.59 | 11.13 | | 26.23 | -7.34 | 12.47 | -527.29 | 337.31 | 113.45 | 22.00 | 18.90 |
| $\left[>N<\right]^{+}$ | 1 | 16.78 | 85.54 | | -210.44 | 166.33 | -63.80 | -105.70 | 301.46 | 90.53 | 8.34 | 20.74 |
| $Cl$ | 1 | 4.39 | 17.68 | | 68.23 | -16.50 | -92.23 | -88.93 | 254.11 | -12.96 | 11.69 | 19.83 |
| Mw (g/mol) |  | $\sum_{i=1}^{5} k_{i}(\Delta\rho_{_{1,i}}^{G})$ | $\sum_{i=1}^{5} k_{i}(\Delta\rho_{_{2,i}}^{G})$ | | $\sum_{i=1}^{5} k_{i}(\Delta n_{_{1,i}}^{G})$ | $\sum_{i=1}^{5} k_{i}(\Delta n_{_{2,i}}^{G})$ | $\sum_{i=1}^{5} k_{i}(\Delta Cp_{_{1,i}}^{G})$ | $\sum_{i=1}^{5} k_{i}(\Delta Cp_{_{2,i}}^{G})$ | $\sum_{i=1}^{5} k_{i}(\Delta u_{_{1,i}}^{G})$ | $\sum_{i=1}^{5} k_{i}(\Delta u_{_{2,i}}^{G})$ | $\sum_{i=1}^{5} k_{i}(\Delta\sigma_{_{1,i}}^{G})$ | $\sum_{i=1}^{5} k_{i}(\Delta\sigma_{_{2,i}}^{G})$ |
| 139.62 |  | 260.05 | 135.48 | | 402.33 | 171.19 | 115.02 | -1740.76 | 2111.31 | 718.24 | 135.54 | 119.20 |

Table A2. List of the decomposed groups of glycerol as the HBD of the GC models for the various properties.

| Group | *l_i_* | **Density** | | | **Refractive index** | | **Heat capacity** | | **Speed of sound** | | **Surface tension** | |
| --- | --- | --- | --- | --- | --- | --- | --- | --- | --- | --- | --- | --- |
|  |  | $\Delta\rho_{1}^{G}$ | $\Delta\rho_{2}^{G}$ | | $\Delta n_{1}^{G}$ | $\Delta n_{2}^{G}$ | $\Delta Cp_{1}^{G}$ | $\Delta Cp_{2}^{G}$ | $\Delta u_{1}^{G}$ | $\Delta u_{2}^{G}$ | $\Delta\sigma_{1}^{G}$ | $\Delta\sigma_{2}^{G}$ |
| **Without Ring** |  |  | |  |  |  |  |  |  |  |  |  |
| $>CH-$ | 1 | -7.03 | -29.59 | | -40.22 | 107.99 | 139.93 | -22.20 | 174.57 | 47.75 | 17.73 | 17.65 |
| $-CH_{2}-$ | 2 | 41.87 | 8.27 | | 19.02 | 5.80 | 1.13 | 159.25 | 36.98 | 16.26 | 16.77 | 17.40 |
| $OH$ | 3 | -30.59 | 11.13 | | 26.23 | -7.34 | 12.47 | -527.29 | 337.31 | 113.45 | 22.00 | 18.90 |
| Mw (g/mol) |  | $\sum_{i=1}^{3} l_{i}(\Delta\rho_{_{1,i}}^{G})$ | $\sum_{i=1}^{3} l_{i}(\Delta\rho_{_{2,i}}^{G})$ | | $\sum_{i=1}^{3} l_{i}(\Delta n_{_{1,i}}^{G})$ | $\sum_{i=1}^{3} l_{i}(\Delta n_{_{2,i}}^{G})$ | $\sum_{i=1}^{3} l_{i}(\Delta Cp_{_{1,i}}^{G})$ | $\sum_{i=1}^{3} l_{i}(\Delta Cp_{_{2,i}}^{G})$ | $\sum_{i=1}^{3} l_{i}(\Delta u_{_{1,i}}^{G})$ | $\sum_{i=1}^{3} l_{i}(\Delta u_{_{2,i}}^{G})$ | $\sum_{i=1}^{3} l_{i}(\Delta\sigma_{_{1,i}}^{G})$ | $\sum_{i=1}^{3} l_{i}(\Delta\sigma_{_{2,i}}^{G})$ |
| 92.09 |  | -15.06 | 20.34 | | 76.51 | 97.57 | 179.60 | -1285.57 | 1260.46 | 420.62 | 117.27 | 109.15 |

Table A3. List of the decomposed groups of choline chloride as the HBA of the AC models for the various properties.

| **Atom** | *k_i_* | **Density** | | **Refractive index** | | **Heat capacity** | | **Speed of sound** | | **Surface tension** | |
| --- | --- | --- | --- | --- | --- | --- | --- | --- | --- | --- | --- |
|  |  | $\Delta\rho_{1}^{A}$ | $\Delta\rho_{2}^{A}$ | $\Delta n_{1}^{A}$ | $\Delta n_{2}^{A}$ | $\Delta Cp_{1}^{A}$ | $\Delta Cp_{2}^{A}$ | $\Delta u_{1}^{A}$ | $\Delta u_{2}^{A}$ | $\Delta\sigma_{1}^{A}$ | $\Delta\sigma_{2}^{A}$ |
| $H$ | 14 | 11.10 | -23.76 | 27.59 | -79.40 | 0.1043 | 0.0139 | 74.50 | 68.77 | 2.29 | 2.74 |
| $C$ | 5 | 11.59 | 35.41 | -35.99 | 171.55 | 0.3838 | 0.0205 | 732.22 | 633.37 | 21.39 | 20.02 |
| $N$ | 1 | 202.25 | 386.06 | -42.29 | 362.85 | 0.3431 | 0.0002 | 3445.83 | 2924.48 | 19.34 | 16.94 |
| $O$ | 1 | -21.11 | 69.14 | 9.63 | 54.84 | 3.1802 | 0.1541 | 1858.40 | 1589.13 | 5.19 | 3.32 |
| $Cl$ | 1 | -3.12 | 212.74 | -4.52 | -105.19 | 482.0574 | 0.8520 | 416.51 | 155.70 | 18.22 | 1.32 |
| Mw (g/mol) | | $\sum_{i=1}^{5} k_{i}(\Delta\rho_{_{1,i}}^{A})$ | $\sum_{i=1}^{5} k_{i}(\Delta\rho_{_{2,i}}^{A})$ | $\sum_{i=1}^{5} k_{i}(\Delta n_{_{1,i}}^{A})$ | $\sum_{i=1}^{5} k_{i}(\Delta n_{_{2,i}}^{A})$ | $\sum_{i=1}^{5} k_{i}(\Delta Cp_{_{1,i}}^{A})$ | $\sum_{i=1}^{5} k_{i}(\Delta Cp_{_{2,i}}^{A})$ | $\sum_{i=1}^{5} k_{i}(\Delta u_{_{1,i}}^{A})$ | $\sum_{i=1}^{5} k_{i}(\Delta u_{_{2,i}}^{A})$ | $\sum_{i=1}^{5} k_{i}(\Delta\sigma_{_{1,i}}^{A})$ | $\sum_{i=1}^{5} k_{i}(\Delta\sigma_{_{2,i}}^{A})$ |
| 139.62 | | 391.37 | 512.35 | 169.13 | 58.65 | 488.9599 | 1.3034 | 10424.84 | 8798.94 | 181.76 | 160.04 |

Table A4. List of the decomposed groups of glycerol as the HBD of the AC models for the various properties.

| **Atom** | *l_i_* | **Density** | | **Refractive index** | | **Heat capacity** | | **Speed of sound** | | **Surface tension** | |
| --- | --- | --- | --- | --- | --- | --- | --- | --- | --- | --- | --- |
|  |  | $\Delta\rho_{1}^{A}$ | $\Delta\rho_{2}^{A}$ | $\Delta n_{1}^{A}$ | $\Delta n_{2}^{A}$ | $\Delta Cp_{1}^{A}$ | $\Delta Cp_{2}^{A}$ | $\Delta u_{1}^{A}$ | $\Delta u_{2}^{A}$ | $\Delta\sigma_{1}^{A}$ | $\Delta\sigma_{2}^{A}$ |
| $H$ | 8 | 11.10 | -23.76 | 27.59 | -79.40 | 0.1043 | 0.0139 | 74.50 | 68.77 | 2.29 | 2.74 |
| $C$ | 3 | 11.59 | 35.41 | -35.99 | 171.55 | 0.3838 | 0.0205 | 732.22 | 633.37 | 21.39 | 20.02 |
| $O$ | 3 | -21.11 | 69.14 | 9.63 | 54.84 | 3.1802 | 0.1541 | 1858.40 | 1589.13 | 5.19 | 3.32 |
| Mw (g/mol) | | $\sum_{i=1}^{3} l_{i}(\Delta\rho_{_{1,i}}^{A})$ | $\sum_{i=1}^{3} l_{i}(\Delta\rho_{_{2,i}}^{A})$ | $\sum_{i=1}^{3} l_{i}(\Delta n_{_{1,i}}^{A})$ | $\sum_{i=1}^{3} l_{i}(\Delta n_{_{2,i}}^{A})$ | $\sum_{i=1}^{3} l_{i}(\Delta Cp_{_{1,i}}^{A})$ | $\sum_{i=1}^{3} l_{i}(\Delta Cp_{_{2,i}}^{A})$ | $\sum_{i=1}^{3} l_{i}(\Delta u_{_{1,i}}^{A})$ | $\sum_{i=1}^{3} l_{i}(\Delta u_{_{2,i}}^{A})$ | $\sum_{i=1}^{3} l_{i}(\Delta\sigma_{_{1,i}}^{A})$ | $\sum_{i=1}^{3} l_{i}(\Delta\sigma_{_{2,i}}^{A})$ |
| 92.09 | | 60.24 | 123.57 | 141.64 | 43.97 | 11.5264 | 0.6350 | 8367.86 | 7217.66 | 98.06 | 91.94 |

The corresponding summations for each physical property can then be used in Eqs. 2-16 for the GC models and Eqs. 17-31 for the AC models. The molecular weight of the DES, also required in these equations, which is 107.93 g/mol for 1 ChCl: 2 Glycerol and 103.97 g/mol for 1 ChCl: 3 Glycerol. Table A5 and A6 present the calculation procedures for the GC and AC models, respectively for 1 ChCl: 2 Glycerol. Also Tables A7 and A8 present the calculation procedures for the GC and AC models, respectively for 1 ChCl: 3 Glycerol.

Table A5. Calculation procedures for calculating density, refractive index, heat capacity, speed of sound and surface tension for the GC models for 1 ChCl: 2 Glycerol.

| **Property** | **GC models** |  |
| --- | --- | --- |
| Density (g/cm^3^) | $\rho_{_{1}}^{G}=1(260.05)+2(-15.06)=229.93$ | (A1) |
|  | $\rho_{_{2}}^{G}=1(135.48)+2(20.34)=176.16$ | (A2) |
|  | $\rho^{G}=\left(\frac{229.93}{107.93}\right)^{-0.2045}+\left(\frac{176.16}{107.93}\right)298.15^{-0.6785}+0.2818$ | (A3) |
|  |  |  |
| Refractive index | $n_{_{1}}^{G}=1(402.33)+2(76.51)=555.35$ | (A4) |
|  | $n_{_{2}}^{G}=1(171.19)+2(97.57)=366.19$ | (A5) |
|  | $n^{G}=\left(555.35\right)^{-0.3597}+\left(366.19\right)298.15^{-1.8254}+1.3695$ | (A6) |
|  |  |  |
| Heat capacity (J/mol.K) | $Cp_{_{1}}^{G}=1(115.02)+2(179.60)=472.22$ | (A7) |
|  | $Cp_{_{2}}^{G}=1(-1740.76)+2(-1285.57)=-4311.90$ | (A8) |
|  | $Cp^{G}=\left(472.22\right)^{0.8653}+\left(-4311.90\right)298.15^{-0.4528}+341.4081$ | (A9) |
|  |  |  |
| Speed of sound (m/s) | $u_{_{1}}^{G}=1(2111.31)+2(1260.46)=4632.23$ | (A10) |
|  | $u_{_{2}}^{G}=1(718.24)+2(420.62)=1559.48$ | (A11) |
|  | $u^{G}=\left(4623.23\right)-\left(1559.48\right)298.15^{0.1851}+1829.8799$ | (A12) |
|  |  |  |
| Surface tension (mN/m) | $\sigma_{_{1}}^{G}=1(135.54)+2(117.27)=370.08$ | (A13) |
|  | $\sigma_{_{2}}^{G}=1(119.20)+2(109.15)=337.50$ | (A14) |
|  | $\sigma^{G}=\left(370.08\right)-\left(337.50\right)298.15^{0.0115}+40.8235$ | (A15) |

Table A6. Calculation procedures for calculating density, refractive index, heat capacity, speed of sound and surface tension for the AC models for 1 ChCl: 2 Glycerol.

| **Property** | **AC models** |  |
| --- | --- | --- |
| Density (g/cm^3^) | $\rho_{_{1}}^{A}=1(391.37)+2(60.24)=511.85$ | (A16) |
|  | $\rho_{_{2}}^{A}=1(512.35)+2(123.57)=759.49$ | (A17) |
|  | $\rho^{A}=\left(\frac{511.85}{107.93}\right)^{-0.4093}+\left(\frac{759.49}{107.93}\right)298.15^{-0.7434}+0.5139$ | (A18) |
|  |  |  |
| Refractive index | $n_{_{1}}^{A}=1(169.13)+2(141.67)=452.47$ | (A19) |
|  | $n_{_{2}}^{A}=1(58.65)+2(43.97)=146.59$ | (A20) |
|  | $n^{A}=\left(452.47\times107.93\right)^{-0.2975}+\left(146.59\times107.93\right)298.15^{-2.9213}+1.4335$ | (A21) |
|  |  |  |
| Heat capacity (J/mol.K) | $Cp_{_{1}}^{A}=1(488.9599)+2(11.5264)=512.0127$ | (A22) |
|  | $Cp_{_{2}}^{A}=1(1.3034)+2(0.6350)=2.5734$ | (A23) |
|  | $Cp^{A}=\left(512.0127\right)^{0.5592}+\left(2.5734\right)298.15^{0.7325}+31.7092$ | (A24) |
|  |  |  |
| Speed of sound (m/s) | $u_{_{1}}^{A}=1(10424.84)+2(8367.86)=27160.56$ | (A25) |
|  | $u_{_{2}}^{A}=1(8798.94)+2(7217.66)=23234.26$ | (A26) |
|  | $u^{A}=\left(27160.56\right)-\left(23234.26\right)298.15^{0.0258}+1607.4690$ | (A27) |
|  |  |  |
| Surface tension (mN/m) | $\sigma_{_{1}}^{A}=1(181.76)+2(98.06)=377.88$ | (A28) |
|  | $\sigma_{_{1}}^{A}=1(160.04)+2(91.94)=343.92$ | (A29) |
|  | $\sigma^{A}=\left(377.88\right)-\left(343.92\right)298.15^{0.0099}+40.4052$ | (A30) |

Table A7. Calculation procedures for calculating density, refractive index, heat capacity, speed of sound and surface tension for the GC models for 1 ChCl: 3 Glycerol.

| **Property** | **GC models** |  |
| --- | --- | --- |
| Density (g/cm^3^) | $\rho_{_{1}}^{G}=1(260.05)+3(-15.06)=214.87$ | (A1) |
|  | $\rho_{_{2}}^{G}=1(135.48)+3(20.34)=196.50$ | (A2) |
|  | $\rho^{G}=\left(\frac{214.87}{103.97}\right)^{-0.2045}+\left(\frac{196.50}{103.97}\right)298.15^{-0.6785}+0.2818$ | (A3) |
|  |  |  |
| Refractive index | $n_{_{1}}^{G}=1(402.33)+3(76.51)=631.86$ | (A4) |
|  | $n_{_{2}}^{G}=1(171.19)+3(97.57)=463.90$ | (A5) |
|  | $n^{G}=\left(631.86\right)^{-0.3597}+\left(463.90\right)298.15^{-1.8254}+1.3695$ | (A6) |
|  |  |  |
| Heat capacity (J/mol.K) | $Cp_{_{1}}^{G}=1(115.02)+3(179.60)=653.82$ | (A7) |
|  | $Cp_{_{2}}^{G}=1(-1740.76)+3(-1285.57)=-5597.47$ | (A8) |
|  | $Cp^{G}=\left(653.82\right)^{0.8653}+\left(-5597.47\right)298.15^{-0.4528}+341.4081$ | (A9) |
|  |  |  |
| Speed of sound (m/s) | $u_{_{1}}^{G}=1(2111.31)+3(1260.46)=5892.69$ | (A10) |
|  | $u_{_{2}}^{G}=1(718.24)+3(420.62)=1980.10$ | (A11) |
|  | $u^{G}=\left(5892.69\right)-\left(1980.10\right)298.15^{0.1851}+1829.8799$ | (A12) |
|  |  |  |
| Surface tension (mN/m) | $\sigma_{_{1}}^{G}=1(135.54)+3(117.27)=487.35$ | (A13) |
|  | $\sigma_{_{2}}^{G}=1(119.20)+3(109.15)=446.65$ | (A14) |
|  | $\sigma^{G}=\left(487.35\right)-\left(446.65\right)298.15^{0.0115}+40.8235$ | (A15) |

Table A8. Calculation procedures for calculating density, refractive index, heat capacity, speed of sound and surface tension for the AC models for 1 ChCl: 3 Glycerol.

| **Property** | **AC models** |  |
| --- | --- | --- |
| Density (g/cm^3^) | $\rho_{_{1}}^{A}=1(391.37)+3(60.24)=572.09$ | (A16) |
|  | $\rho_{_{2}}^{A}=1(512.35)+3(123.57)=883.06$ | (A17) |
|  | $\rho^{A}=\left(\frac{572.09}{103.97}\right)^{-0.4093}+\left(\frac{883.06}{103.97}\right)298.15^{-0.7434}+0.5139$ | (A18) |
|  |  |  |
| Refractive index | $n_{_{1}}^{A}=1(169.13)+3(141.67)=594.14$ | (A19) |
|  | $n_{_{2}}^{A}=1(58.65)+3(43.97)=190.56$ | (A20) |
|  | $n^{A}=\left(594.14\times107.93\right)^{-0.2975}+\left(190.56\times107.93\right)298.15^{-2.9213}+1.4335$ | (A21) |
|  |  |  |
| Heat capacity (J/mol.K) | $Cp_{_{1}}^{A}=1(488.9599)+3(11.5264)=523.5391$ | (A22) |
|  | $Cp_{_{2}}^{A}=1(1.3034)+3(0.6350)=3.2084$ | (A23) |
|  | $Cp^{A}=\left(523.5391\right)^{0.5592}+\left(3.2084\right)298.15^{0.7325}+31.7092$ | (A24) |
|  |  |  |
| Speed of sound (m/s) | $u_{_{1}}^{A}=1(10424.84)+3(8367.86)=35528.42$ | (A25) |
|  | $u_{_{2}}^{A}=1(8798.94)+3(7217.66)=30451.92$ | (A26) |
|  | $u^{A}=\left(35528.42\right)-\left(30451.92\right)298.15^{0.0258}+1607.4690$ | (A27) |
|  |  |  |
| Surface tension (mN/m) | $\sigma_{_{1}}^{A}=1(181.76)+3(98.06)=475.94$ | (A28) |
|  | $\sigma_{_{1}}^{A}=1(160.04)+3(91.94)=435.86$ | (A29) |
|  | $\sigma^{A}=\left(475.94\right)-\left(435.86\right)298.15^{0.0099}+40.4052$ | (A30) |

Table A9 shows the final results for the GC and AC models for each physical property, together with the corresponding *AARD%* with respect to the experimental values for both the 1 ChCl: 2 Glycerol and 1 ChCl: 3 Glycerol DESs.

Table A9. The list of calculated values by the GC and AC models for each investigated physical property, with the corresponding *AARD%* for 1 ChCl: 2 Glycerol and 1 ChCl: 3 Glycerol.

| Property | Experimental value | Calculated value by model | | AARD% | |
| --- | --- | --- | --- | --- | --- |
|  |  | GC | AC | GC | AC |
|  | 1 ChCl: 2 Glycerol | | | | |
| Density (g/cm^3^) | 1.1912 | 1.1727 | 1.1445 | 1.55 | 3.92 |
| Refractive index | 1.48675 | 1.48362 | 1.4747 | 0.21 | 0.81 |
| Heat capacity (J/mol.K) | 218.5 | 220.7 | 231.6 | 1.01 | 6.00 |
| Speed of sound (m/s) | 2001 | 1976 | 1855 | 1.25 | 7.30 |
| Surface tension (mN/m) | 57.24 | 50.55 | 54.41 | 11.69 | 4.94 |
|  | 1 ChCl: 3 Glycerol | | | | |
| Density (g/cm^3^) | 1.2030 | 1.1834 | 1.1344 | 1.63 | 5.70 |
| Refractive index | 1.48514 | 1.4819 | 1.4719 | 0.22 | 0.89 |
| Heat capacity (J/mol.K) | - | 190.2 | 273.2 | - | - |
| Speed of sound (m/s) | - | 2037.9 | 1861.8 | - | - |
| Surface tension (mN/m) | - | 51.28 | 55.19 | - | - |

1. Corresponding author at: LAQV, REQUIMTE, Departamento de Química da Faculdade de Ciências e Tecnologia, Universidade Nova de Lisboa, 2829-516 Caparica, Portugal.

   Tel.: +352 294 96 80

   E-mail address: ard08968@fct.unl.pt (Ana Rita C. Duarte). [↑](#footnote-ref-1)
